# A patient-derived mutation of epilepsy-linked *LGI1* increases seizure susceptibility through regulating K_v_1.1

**DOI:** 10.1186/s13578-023-00983-y

**Published:** 2023-02-20

**Authors:** Lin Zhou, Kang Wang, Yuxiang Xu, Bin-Bin Dong, Deng-Chang Wu, Zhao-Xiang Wang, Xin-Tai Wang, Xin-Yu Cai, Jin-Tao Yang, Rui Zheng, Wei Chen, Ying Shen, Jian-She Wei

**Affiliations:** 1 Department of Physiology and Department of Psychiatry, Sir Run Run Shaw Hospital, Zhejiang University School of Medicine, Hangzhou, 310020 China; 2 Department of Neurology, First Affiliated Hospital of Zhejiang University School of Medicine, Hangzhou, 310003 China; 3 School of Life Sciences, Henan University, Kaifeng, 475004 China

**Keywords:** ADLTE, Epilepsy, Leucine-rich glioma inactivated 1, Precision medicine

## Abstract

**Background:**

Autosomal dominant lateral temporal epilepsy (ADLTE) is an inherited syndrome caused by mutations in the leucine-rich glioma inactivated 1 (LGI1) gene. It is known that functional LGI1 is secreted by excitatory neurons, GABAergic interneurons, and astrocytes, and regulates AMPA-type glutamate receptor-mediated synaptic transmission by binding ADAM22 and ADAM23. However, > 40 LGI1 mutations have been reported in familial ADLTE patients, more than half of which are secretion-defective. How these secretion-defective LGI1 mutations lead to epilepsy is unknown.

**Results:**

We identified a novel secretion-defective LGI1 mutation from a Chinese ADLTE family, LGI1-W183R. We specifically expressed mutant LGI1^W183R^ in excitatory neurons lacking natural LGI1, and found that this mutation downregulated K_v_1.1 activity, led to neuronal hyperexcitability and irregular spiking, and increased epilepsy susceptibility in mice. Further analysis revealed that restoring K_v_1.1 in excitatory neurons rescued the defect of spiking capacity, improved epilepsy susceptibility, and prolonged the life-span of mice.

**Conclusions:**

These results describe a role of secretion-defective LGI1 in maintaining neuronal excitability and reveal a new mechanism in the pathology of LGI1 mutation-related epilepsy.

**Supplementary Information:**

The online version contains supplementary material available at 10.1186/s13578-023-00983-y.

## Introduction

Epilepsy, characterized by recurrent seizures, is a common brain disorder that affects 1–2% of the population [1, 2]. At present, the therapeutic effect of antiepileptic drugs is not satisfactory. To develop novel therapeutic targets, it is essential to clarify the molecular mechanisms of seizure genesis. Autosomal dominant lateral temporal epilepsy (ADLTE) is an inherited syndrome caused by mutations in the leucine-rich glioma inactivated 1 (LGI1) gene, which encodes a secreted protein [3–12]. Haploinsufficiency of LGI1 has been suggested to be the major pathogenic basis for human ADLTE [4, 13, 14]. In mouse models, the global knockout of LGI1 results in generalized seizures and premature death [15–17], and the heterozygous knockout of LGI1 causes increased seizure susceptibility to either acoustic stimuli [15] or the convulsant agent pentylenetetrazole (PTZ) [16, 18].

It has been shown that functional LGI1 exists in excitatory neurons, interneurons, astrocytes, and oligodendrocytes [19, 20], and regulates the activity of K_v_1 [18, 21–23] and AMPA-type glutamate receptor-mediated synaptic transmissions through binding to a distintegrin and metalloproteinases (ADAMs) [24, 25]. Furthermore, enhancing the interaction between the LGI1-ADAM22 complex and PSD-95 family proteins may prevent epilepsy [26–29], and restoring K_v_1 activity using celecoxib, a drug approved by the Food and Drug Administration, ameliorates seizure susceptibility in global LGI1 knockout mice [18]. Although these strategies might be effective to reduce epileptic seizures in LGI1 knockout mice, clinical situation is much more complicated: > 40 *LGI1* mutations have been found in patients with familial ADLTE [3–12], highlighting the importance of the gene-mutation-cell-behavior relationship on an individual basis. However, how these LGI1 mutations lead to different disease phenotypes in ADLTE patients remains unclear. For example, 41 missense mutations of *LGI1* yield either secretion-defective or -competent proteins [3–12, 26, 28], but the functions of the secretion-defective proteins, which cannot act on synaptic transmission as the extracellular binding partner of ADAM22/ADAM23, are almost unknown. It has been shown that LGI1 can act as a cytosolic protein to inhibit the inactivation of presynaptic K_v_1 [21], and that LGI1 and K_v_1 coexist in axonal initial segment [22]. Accordingly, we hypothesize that secretion-defective LGI1 proteins may act on K_v_1 to regulate neuronal excitability.

Here, we identified a novel missense *LGI1* mutation, p.Trp183Arg, from a Chinese ADLTE pedigree. To investigate the function of this mutant in the central nervous system (CNS), we used Cre-Loxp-based viral infection to specifically express mutant LGI1^W183R^ in mouse excitatory neurons that lacked native LGI1. Utilizing in vivo and in situ electrophysiological recordings and computer simulation, we found that the LGI1^W183R^ mutation produced secretion-defective LGI1 protein, which downregulated K_v_1.1 activity, caused neuronal hyperexcitability and firing irregularity, and increased seizure susceptibility. Moreover, restoring K_v_1.1 in excitatory neurons was able to correct the deficits in firing and ameliorate seizure susceptibility in mice.

## Materials and methods

### Clinical information

The proband, a 16-year-old man, came to the hospital due to repeated unconsciousness and generalized convulsions. The detailed clinical information of the proband and his family is shown in Additional files 11 and 12 (*Proband clinical information* and *Proband family sequencing result*). In brief, the form of his attacks was short-lived buzzing and tinnitus, followed by loss of consciousness, stiffness of the limbs, and foaming at the mouth, which lasted for ~ 2 min. He also suffered from isolated attacks of tinnitus, which occurred on average once a month. He was delivered naturally at full term, and the birth process was smooth, and his developmental milestones were normal. Physical examination of his nervous system was normal as well: MRI of his head showed no evident abnormality. However, video EEG monitoring showed occasional epileptiform discharges in the right temporal region. Initial treatment was valproic acid (0.5 g) twice a day, but he still had seizures. Then oxcarbazepine (0.3 g) was added twice a day, and the proband remained free of attacks for 4 years. The proband’s father had similar seizures at the age of 18, took carbamazepine (0.2 g) twice a day, and had no attacks for ~ 20 years. The proband’s grandfather had a grand mal seizure at the age of 19 and often auditory aura since that time, but did not take antiepileptic drugs, thereby living with seizures every 3–5 years.

### Patient EEG recording

A 20-h EEG dataset was collected using a Nicolet V32 EEG monitor (Natus Neurology Inc.) with 21 electrodes according to the international 10–20 system. During recording, the Fz electrode served as the common reference. EEG signals were sampled at 256 Hz and bandpass-filtered at 0.01–100 Hz. To guarantee data quality, the impedance of all electrodes was calibrated at < 5 KΩ before acquisition. During EEG recording, one camera was used to monitor the behavior of the patient. The data were read and analyzed by two epileptologists independently.

### Animals

*LGI1*^flox/flox^ mice (Loxp-flanked encompassing exons 5 and 7) were created at the Model Animal Research Center of Nanjing University. All mice were maintained at the Experimental Animal Center of Zhejiang University and kept in temperature-controlled conditions under a 12:12 h light/dark cycle with food and water ad libitum. Male mice were used in all experiments.

### Antibodies and reagents

Following antibodies and reagents were used: anti-NeuN (#MAB377) and anti-GAPDH (#MAB374) from Millipore, DTx-K (#ab141795) and anti-LGI1 (#ab30866) from Abcam, anti-CaMKIIα (#611292) from Bio-rad, anti-Iba1 (#SAG4318) from Wako, anti-GFAP (#Z0334) from DAKO, anti-PV (#235) from SWAT, anti-Flag (#T20008) from Abmart, and Goat anti-mouse/rabbit IgG horseradish peroxidase-conjugated antibodies (#31446 and #31460) from Thermo Fisher Scientific. Other reagents were from Sigma or Tocris unless stated otherwise.

### Cell culture and DNA transfection

HEK293 cells were cultured in DMEM and supplemented with 10% FBS in an incubator (95% O_2_/5% CO_2_; 37 °C). Cells were transfected in OPTI-MEM with plasmids using lipofectamine 2000. Cells were treated with CHX (50 μM) to inhibit protein synthesis at 24, 32, 40 and 44 h after the transfection [30]. Cells were harvested 48 h after the transfection and lysed in RIPA buffer (1% Triton X-100, 0.5% deoxycholate, 0.2% SDS, 100 mM NaCl, 1 mM EDTA, 50 mM Tris–HCl; pH 7.4) plus protease inhibitors and centrifuged at 10,000 × *g* at 4 °C for 20 min to collect supernatant fractions.

For Co-IP experiments, HEK293 cells were harvested 48 h after transfection with Flag-LGI1^WT^ or Flag-LGI1^W183R^, lysed in RIPA buffer plus the protease inhibitor and centrifuged at 10,000 × *g* at 4 °C for 20 min to collect supernatant fractions. These fractions were incubated with rabbit anti-Flag antibody, which was pre-coupled to protein A-sepharose beads for 2 h at 4 °C. Proteins on the beads were washed 3 times with 50 mM Tris–HCl and extracted with 2 × SDS sample buffer. Protein samples were immunoblotted with mouse ubiquitin antibody.

### Western blotting

Samples were rinsed with PBS and diluted in a 1% SDS-containing protease inhibitor mixture. After determining the protein concentration using BCA protein assay, equal quantities of protein were loaded and fractionated on SDS-PAGE gels, transferred to PVDF membranes, immunoblotted with antibodies, and visualized by enhanced chemiluminescence. The primary antibody dilutions were 1:1000 for LGI1 and 1:10,000 for GAPDH. The secondary antibodies were anti-rabbit (1:10,000) and anti-mouse (1:10,000). Film signals were digitally scanned and protein levels were quantified by measuring the integrated optical densities of the bands after background subtraction using ImageJ 1.42q (NIH).

### RNA preparation and real-time PCR

mRNA levels were assessed by real-time PCR using an ABI PRISM 7500 sequence detection system (Applied Biosystems). cDNA was synthesized by reverse transcription using oligo (dT) as the primer and proceeded to real-time PCR with gene-specific primers in the presence of SYBR Premix Ex Taq. Quantification was performed by the comparative cycle threshold (Ct) method, using GAPDH as the internal control. Following forward (F) and reverse (R) primers were used to amplify: *LGI1*-F: 5ʹ-GCT GCA GCT CTT GTT ATT TAC GTC G-3ʹ, and *LGI1*-R: 5ʹ-GAG CCA TTC CAC CAG CCA CTT CAA C-3ʹ. *GAPDH*-F: 5′-TGT TAC CAA CTG GGA CGA CA-3′, and *GAPDH*-R: 5′-AAG GAA GGC TGG AAA AGA GC-3′.

### Immunohistochemistry

Coronal cortical sections were cut at 25 m and placed in a blocking solution (1% BSA, 0.3% triton, 10% normal goat serum) for 1 h at RT. After washing with PBS, the sections were incubated with primary antibodies overnight at 4 °C and then incubated with secondary antibody for 1 h at RT. These sections were then mounted using ProLong Gold Antifade reagent with DAPI. The antibody dilutions were 1:250 (CaMKIIα), 1:500 (Iba1 and GFAP), 1:1000 (NeuN, Alexa Fluor 594/647-conjugated goat anti-rabbit IgG, and 594/647-conjugated goat anti-mouse IgG), and 1:2000 (PV). All antibodies were diluted in PBS containing 1% BSA and 1% normal goat serum.

### Intracranial injection

P0 pups were cryoanesthetized at − 20 °C for 2–3 min before injection. A solution of AAV containing 0.05% trypan blue was injected bilaterally into the ventricles using a 10-μl syringe with a 32-gauge needle (Hamilton). The injection site was located 2/5 of the distance along a line defined between each eye and the lambda intersection of the skull at a depth of 3 mm. Viral solution (2 l) was injected into each lateral ventricle (AAV final unit at 1 × 10^^13^ viral genomes/ml). After injections, pups were placed on a warming pad, and then returned to their mothers for care [31].

### PTZ-induced seizures

Seizures were measured in mice (P35) injected with PTZ at 45 mg/kg (i.p.). The seizure activity was observed and scored by investigators who were blinded to the genotype throughout the experiments. Seizures were scored for 30 min after the injection as follows [18]: 1, hypoactivity (abdomen in full contact with the bottom of the cage in the resting position); 2, focal clonus (of face, head, or forelimbs); 3, generalized clonus (rearing, falling, and clonus of four limbs and tail); and 4, clonic (tonic seizure, tonic hindlimb extension, or death).

### Mouse EEG recording

Mice were deeply anesthetized with pentobarbital (30 mg/kg) and placed in a stereotaxic apparatus (Stoelting). Recording electrodes (#795500, A. M. Systems) made of twisted stainless-steel wires (diameter: 0.125 mm) insulated with Teflon were implanted into the hippocampal CA1 region (in mm; bregma: − 2.0; mediolateral: ± 2.0; dorsoventral: − 1.5) according to the Paxinos and Franklin mouse brain atlas [32]. Two screws were placed in the skull over the cerebellum to serve as the reference and ground electrodes. The maximal tip separation between recording and reference electrodes was 0.5 mm. After complete recovery from the surgery, the mice were placed in a transparent cage and allowed to move freely. EEG signals were band-pass filtered spanning DC to 200 Hz and sampled at 2 kHz using an amplifier (Neuroscan System). EEG recordings were continued for 30 min after PTZ injection [18, 33].

### In vitro electrophysiology

Coronal slices of the hippocampus (300 μm) from P17–20 mice were cut in ice-cold aCSF (in mM: 125 NaCl, 3 KCl, 1.25 NaH_2_PO_4_, 2 MgSO_4_, 2 CaCl_2_, 25 NaHCO_3_, 10 glucose) on a vibrating tissue slicer (VT 1000S, Leica). After recovery for 30 min at 37 °C, the slices were incubated at room temperature (RT) for 60 min and then transferred to the recording chamber and superfused at 2 ml/min with aCSF at RT. All solutions were saturated with 95% O_2_/5% CO_2_.

Neurons were visualized under an upright microscope (BX51, Olympus) with a 40 × water-immersion objective equipped with infrared differential interference contrast enhancement. Whole-cell recordings were made with an Axon MultiClamp 700B amplifier (Molecular Devices). Glass pipettes (3–5 MΩ) were filled with a solution containing (in mM): 120 K-gluconate, 20 KCl, 10 HEPES, 2 MgCl_2_, 10 Na-phosphocreatine, 4 Mg-ATP, 0.3 Na-GTP, 0.1 EGTA (pH 7.3; 290 Osm). Currents were filtered at 2 kHz and digitized at 10 kHz. Recordings were excluded from analysis if series resistance, input resistance, or holding current varied by 15% over the course of an experiment. All electrophysiological experiments were performed at RT.

Passive neuronal properties were measured from single APs. The rheobase was defined as the minimum depolarizing current needed to elicit an AP. AP amplitude was measured as the voltage difference between peak and resting potentials. The half-width was determined at half of spike amplitude. The input–output relationship between injected currents and spikes was determined when neurons received a series of currents ranging from 20 to 200 pA (20-pA increments) with a duration of 800 ms. The fast AHP was defined as the slope of the 3-ms Vm immediately following the spike [34, 35]. For mEPSC recording, the neurons were held at –70 mV in the presence of TTX (1 μM) and GABAzine (20 μM). To measure K^+^ currents, TTX (1 μM) and CdCl_2_ (100 μM) were added to the aCSF to block Na^+^ and Ca^2+^ channels, and a series of voltage pulses (3 s) from − 70 mV to 40 mV (10-mV increment) were applied under the voltage-clamp configuration. K_v_1.1 activation/inactivation curves were constructed from steady-state currents after the conversion to conductance, normalized to the maximum conductance, and fitted with a single Boltzmann equation [18, 36].

### Non-stationary noise analysis

In order to estimate single-channel current (*I*) and available number of channels (*N*), peak-scaled non-stationary noise analysis was applied to each ensemble of K_v_1.1 current simulated with an activation command pulse, based on previous work [37–40]. The peak of mean current response waveform was scaled to the response value at the corresponding point in time of each individual event [41]. In this case, *N* corresponded to the average number of channels open at the peak. To assign similar weights to all phases of the ensemble mean waveform, from the peak to the end of the decay, the amplitude interval from the peak to the baseline was divided into an equal number of intervals [41, 42]. The amplitude intervals were then translated to the corresponding time intervals, and the variance and mean current were calculated for each interval over all the event waveforms using Excel software (Microsoft). Leak was subtracted off-line with subtraction pulses collected throughout the run. The variance was plotted against the mean current and the data points were fitted with a parabolic function [37], omitting the rising phase of the response: σ^2^(*I*) = *iI* − *I*^2^/N, where for any given potential, σ^2^ was the variance, *i* was the single channel current, *I* was the macroscopic mean current, and N was the number of channels. The values for N and *i* were then estimated from the fit of the variance *vs* mean data to the equation.

### Computer simulation

The simulation was carried out using NEURON v7.5 software. In the three-dimensional computational model of a CA1 pyramidal neuron [43, 44], uniform passive parameters (τ_m_ = 28 ms; C_m_ = 0.75 μF/cm^2^; R_m_ = 37.3 KΩ/cm^2^) were set for the entire neuron and RMP was set at − 65 mV. The kinetics and distribution of active somatic, dendritic and axonal channels, including *I*_Na_, *I*_DR_ (delayed rectifier K^+^), *I*_A_ (transient K^+^), *I*_KM_ (M K^+^), K_v_1.1, and *I*_h_ (non-selective hyperpolarization-activated channels), were defined according to data available for CA1 pyramidal neurons [45]. The conductance of low-threshold Ca^2+^ and Ca^2+^-dependent K^+^ and Ca^2+^ extrusion machinery was also included in the neuronal model. The densities of *I*_Na_ and *I*_DR_ were set to 450 and 200 pS/μm^2^, respectively, at the soma and apical dendrites, and their density in axons was increased threefold. The somatic density of *I*_A_ and *I*_h_ were 300 and 0.1 pS/μm^2^, respectively, and they were increased linearly with distance from the soma. *I*_KM_ was inserted into the soma at a density of 60 pS/μm^2^ and into the axon at a fourfold higher density. The conductance of Ca^2+^ and Ca^2+^-dependent K^+^ channels was set to 1 and 0.1 pS/μm^2^, respectively. K_v_1.1 was set at a uniform density of 100 pS/μm^2^ in both the soma and the axon [46]. The steady-state activation curve and time constants for K_v_1.1 (V_1/2_ = − 30 mV) were defined according to previous work [47].

### Statistical analysis

Data were analyzed using GraphPad Prism 8.0 (GraphPad Software) and Igor Pro 6.0 (Wavemetrics). Statistical differences were determined using the unpaired *t* test with Welch's correction for two-group comparisons or 2-way ANOVA followed by Bonferroni’s post hoc test for multiple comparisons. Survival curves were analyzed by Kaplan–Meier survival estimate using the log-rank test. The accepted level of significance was *P* < 0.05. *n* represents the number of animals or cells. Data in the text and figures are presented as the mean ± SEM.

## Results

### A novel missense LGI1 mutation produces a secretion-defective LGI1 protein

**Fig.1:**
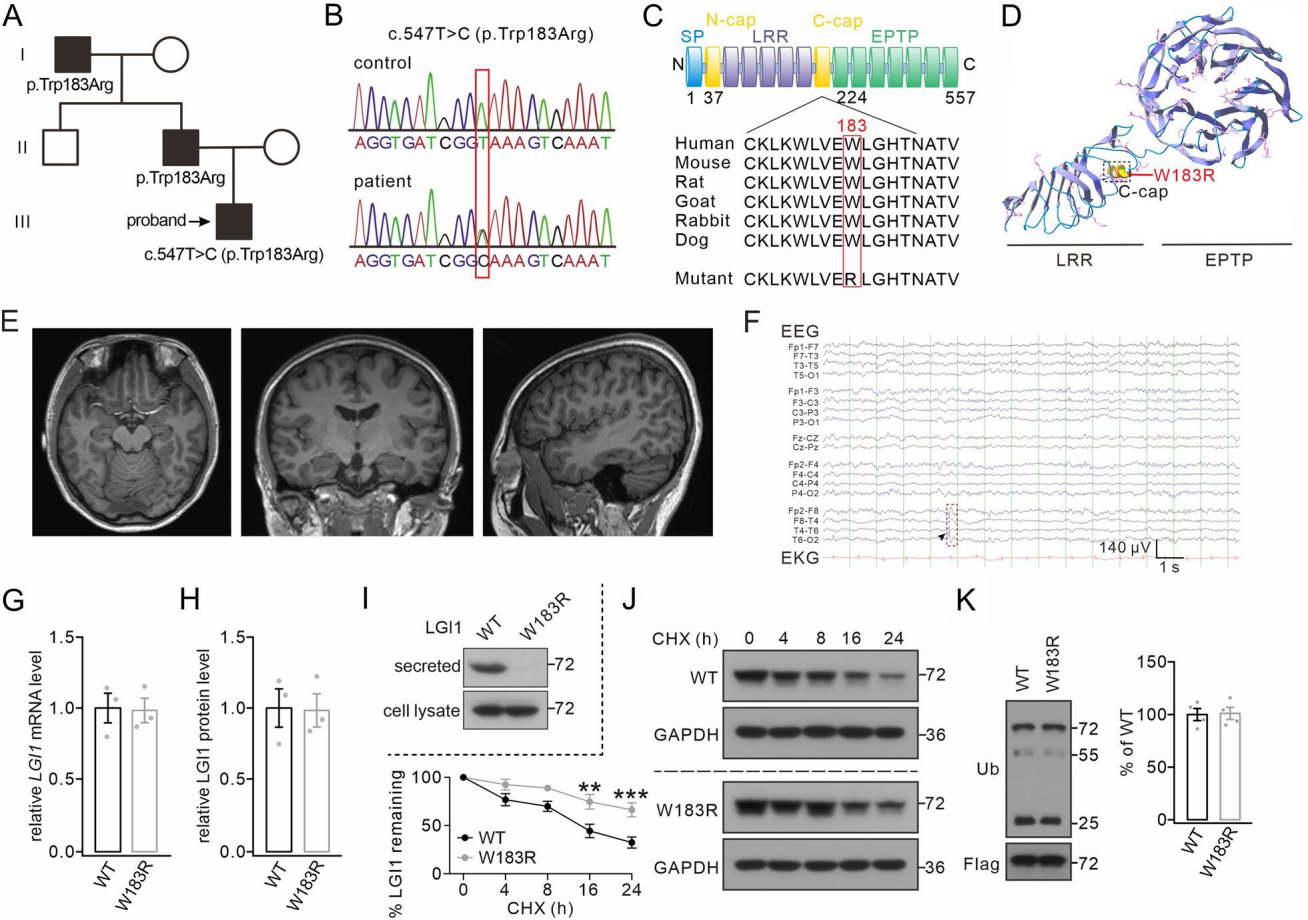
Genetic and expression analysis of the LGI1^W183R^ mutation. **A** Pedigree of the LGI1^W183R^ variant. (square, male, circles, females; see Additional file for details). **B** Chromatograms of c.547 T > C within *LGI1*. (upper, normal sequence; lower, mutant sequence). **C** Upper, domains within the LGI1 protein: N-cap, LRR, C-cap, and EPTP. The LGI1^W183R^ mutation maps onto the C-cap domain. Lower, alignment analysis of LGI1 orthologues in different vertebrate species shows the conservation of the W183 residue. **D** Mapping of the LGI1^W183R^ mutation on the 3D structure of LGI1. **E** Brain MRIs of the proband showing normal brain structure (horizontal, coronal, and sagittal views from left to right). **F** Representative EEG recording from the proband (arrowhead, an epileptiform discharge during the interictal period; left electrodes in the Bipolar Montage. **G** mRNA levels of LGI1 in HEK cells transfected with LGI1^WT^ or LGI1^W183R^. **H** Total LGI1 protein in cultures transfected with LGI1^WT^ or LGI1^W183R^. **I** LGI1 expression in the medium (secreted) and cell lysates of cultures transfected with LGI1^WT^ or LGI1^W183R^. **J** LGI1 protein levels at annotated time points in HEK cells transfected by LGI1^WT^ or LGI1^W183R^ and continuously treated with CHX. **K** Immunoprecipitation with rabbit anti-Flag antibody followed by western blots using mouse anti-Flag antibody and mouse anti-Flag-ubiquitin show no difference in LGI1 ubiquitination level between the two groups (Flag-LGI1^WT^ or Flag-LGI1^W183R^ transfected into HEK cells). See Additional file 4: Table S1 for statistics. **P < 0.01. ***P < 0.001

We treated a 16-year-old Chinese man who developed recurrent epileptic seizures (see Materials and Methods and Additional file). His episodes were characterized by generalized tonic–clonic seizures preceded by auditory auras. His father and grandfather had similar manifestations from the puberty, strongly indicating ADLTE [5, 48]. We applied whole-exome sequencing using a blood sample of the proband, and identified a novel missense LGI1 variant, c.547 T (p.Trp183Arg) (Fig. 1A, B), which is absent from the ExAC, dbSNP, 1000G, and gnomAD databases. Moreover, both his father and grandfather carried the same heterozygotic variant (Fig. 1A), consistent with a pattern of autosomal dominant inheritance. The Trp183 residue is highly conserved throughout vertebrate species (Fig. 1C), and is located in the C-cap domain of LGI1 (Fig. 1C, D). The brain magnetic resonance imaging (MRI) of the proband appeared normal (Fig. 1E). The drug treatment we provided effectively controlled his seizures (see Additional file), and thereby only brief and occasional interictal epileptiform discharges were detected after the treatment (Fig. 1F).

To characterize this missense mutation, we generated plasmids encoding wild-type LGI1 (LGI1^WT^) or mutant LGI1^W183R^, and transfected them into HEK cells. mRNA analysis revealed no difference in the level of transcripts between LGI1^W183R^ and LGI1^WT^ (Fig. 1G). Western blots with anti-LGI1 antibody also showed no difference in the protein level between LGI1^W183R^ and LGI1^WT^ (Fig. 1H). It has been shown that some of *LGI1* mutations yield secretion-defective LGI1 proteins [26, 28]. To investigate whether LGI1^W183R^ mutation leads to defective secretion, we measured LGI1 protein in conditioned medium and cell lysates, and found that LGI1^WT^ protein was secreted by HEK cells, whereas LGI1^W183R^ protein was not (Fig. 1I). We compared the stability of LGI1^WT^ and LGI1^W183R^ proteins in the cell lysates using cycloheximide (CHX) treatment. The expressions of both LGI1^WT^ and LGI1^W183R^ proteins were reduced over time during CHX treatment, but the rate of decrease of LGI1^WT^ was much faster than that of LGI1^W183R^ (Fig. 1J), which might be due to the secretion of LGI1^WT^. To investigate whether the LGI1^W183R^ mutation affects LGI1 ubiquitination, we added ubiquitin to HEK cells and found that both LGI1^WT^ and LGI1^W183R^ proteins were ubiquitylated to the same extent, implying that LGI1^W183R^ mutation does not affect the degradation of LGI1 (Fig. 1K).

### Expressing LGI1^W183R^ in excitatory neurons results in epileptic seizures

**Fig.2:**
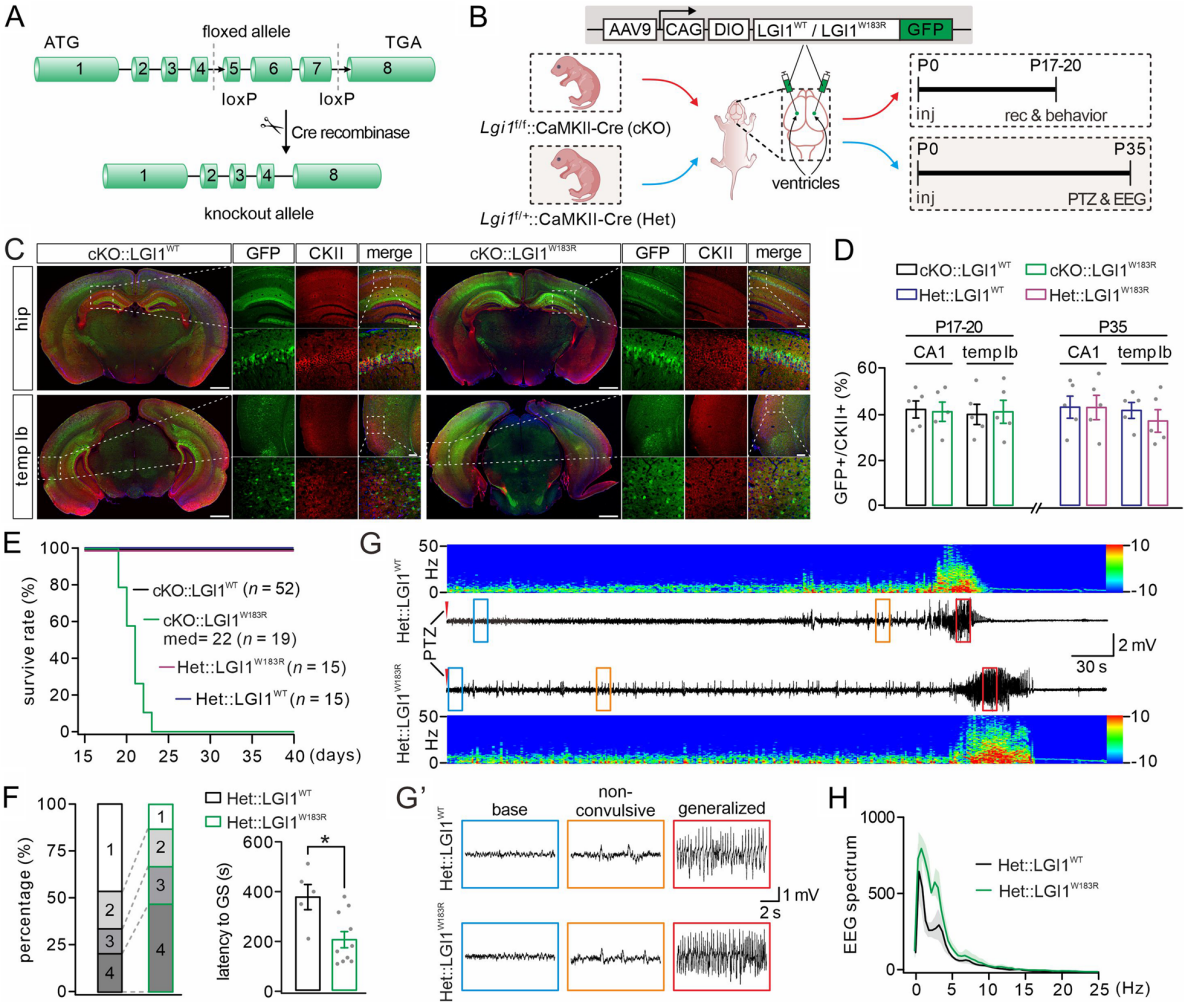
Expressing LGI1^W183R^ in excitatory neurons increases seizure susceptibility. **A** Gene targeting strategy for the generation of LGI1^flox/flox^ mice. **B** AAV9-DIO-LGI1^WT^-GFP or AAV9-DIO-LGI1^W183R^-GFP is expressed in cKO or Het excitatory neurons (CaMKII-Cre). Viruses are bilaterally injected into the ventricles (P0). cKO mice subjected to video observation and electrophysiological recording at P17–20 and Het mice are subjected to PTZ treatment and EEG recording at P35. **C** Representative images for triple fluorescence, GFP, CaMKII(CKII) and DAPI, in the hippocampus (hip) and temporal cortex (temp lb) of cKO mice (P17) (scale bars: 1 mm (whole brain); 50 μm (magnified). **D** Number ratios of GFP + *vs* CaMKII-Cre + cells in cKO and Het mice expressing LGI1^WT^ or LGI1^W183R^ (*n* = 5 per group). At P17–20, the ratio of GFP + *vs* CaMKII + is 43 ± 4 (CA1; LGI1^WT^) and 42 ± 4 (CA1; LGI1^W183R^), *P* = 0.86; 41 ± 4 (temp lb; LGI1^WT^) and 42 ± 5 (temp lb; LGI1^W183R^), *P* = 0.86. At P35, the ratio of GFP + *vs* CaMKII + is 44 ± 5 (CA1; LGI1^WT^) and 44 ± 5 (CA1; LGI1^W183R^), *P* = 0.98; 42 ± 3 (temp lb; LGI1^WT^) and 38 ± 5 (temp lb; LGI1^W183R^), *P* = 0.47. **E** Kaplan–Meier survival curves. **F** Quantification of reactions to PTZ injection. Latency to generalized seizure (GS): 383 ± 50 s (Het::LGI1^WT^; *n* = 5) and 212 ± 33 s (Het::LGI1^W183R^; *n* = 10), *P* = 0.023. **G** Representative EEGs and power spectral analysis in Het::LGI1^WT^ and Het::LGI1^W183R^ mice (P35) during PTZ-induced seizures. **G** Enlarged view of EEGs in **G**. **H** Spectral analysis of the EEGs. Grey dots indicate individual data points. *P < 0.05

Previous work has shown that the depletion of LGI1 in excitatory neurons is critical to the onset of seizures [19], highlighting the importance of excitatory neurons in ADLTE. Hence, we hypothesized that the pathogenic mechanism of LGI1^W183R^ mutation is dependent of excitatory neurons. To test this idea, we expressed LGI1^W183R^ solely in excitatory neurons in the brain. First, we applied Cre-Loxp technique to generate *LGI1*^flox/+^ mice (Fig. 2A), which were further intercrossed with CaMKII-Cre mice to obtain either CaMKII-Cre;*LGI1*^flox/+^ (Het) or CaMKII-Cre;*LGI1*^flox/flox^ (cKO), where LGI1 was either haploinsufficient or deficient in excitatory neurons (Fig. 2B). Next, we injected AAV9-DIO-LGI1^WT^-GFP or AAV9-DIO-LGI1^W183R^-GFP bilaterally into the ventricles of either Het or cKO mice at P0 (Fig. 2B). With this approach, LGI1^WT^-GFP or LGI1^W183R^-GFP was specifically expressed in excitatory neurons, in which endogenous LGI1 was deleted or haploinsufficient. Finally, cKO or Het mice with exogenous LGI1^WT^ or LGI1^W183R^ were subjected to video monitoring of autonomous or PTZ-induced epileptic seizures and/or electroencephalogram (EEG) recordings (Fig. 2B). With this strategy, the expression of LGI1^WT^ or LGI1^W183R^ was confirmed by confocal imaging of GFP fluorescence and CaMKIIα expression. We found that the GFP signal was broadly expressed in the cerebral cortex and the hippocampus (Fig. 2C). Moreover, GFP signal was well co-localized with CaMKIIα signal (Fig. 2C), indicating the specific expression of LGI1^WT^ and LGI1^W183R^ proteins in excitatory neurons. Counting the numbers of neurons showed no difference in the ratio of GFP-positive neurons among CaMKIIα-positive neurons between the LGI1^WT^ and LGI1^W183R^ groups at two developmental stages, P17–20 and P35 (Fig. 2D). These results indicate that, with our strategy, exogenous LGI1^WT^ and LGI1^W183R^ proteins are robustly expressed in excitatory neurons upon the viral injection of LGI1^WT^ or LGI1^W183R^ into the ventricles. This conclusion was further strengthened by confocal imaging of GFP and the specific marker proteins for other major types of brain cells, including parvalbumin (PV)-positive interneurons, astroglia, and microglia (Additional file 1: Fig. S1).

The loss of LGI1 in excitatory neurons causes epileptic seizures and premature death [19]. Accordingly, we considered whether LGI1^W183R^ protein in excitatory neurons may lead to these phenotypes as well. The majority of cKO::LGI1^W183R^ mice (17/19) died before P21 and their median lifetime was 22 days, as shown in the Kaplan–Meier survival curves (Fig. 2E). In contrast, cKO::LGI1^WT^, Het::LGI1^WT^ and Het::LGI1^W183R^ littermates survived for > 40 days (Fig. 2E).The cKO::LGI1^W183R^ mice often had spontaneous seizures (generalized tonic or clonic seizures) (Additional file 9: Video S1) at an onset age of P16 with a frequency between 0.25 and 1 per hour and a mean duration of 45.5 ± 18.7 s (Additional file 6: Table S2). In contrast, cKO::LGI1^WT^, Het::LGI1^WT^ and Het::LGI1^W183R^ mice displayed no autonomous seizures. Because mutant mice with LGI1 haploinsufficiency display increased seizure susceptibility to PTZ [28], we examined PTZ-induced seizures in Het::LGI1^WT^ and Het::LGI1^W183R^ mice at P35. Our results showed that, compared to Het::LGI1^WT^ mice, seizure severity was significantly greater in Het::LGI1^W183R^ mice, as shown by more frequent generalized seizures: the majority of Het::LGI1^W183R^ mice (10/15) were at stages 3/4 and displayed a shortened latency to generalized seizures, whereas the majority of Het::LGI1^WT^ (10/15) mice were at stages 1/2 (Fig. 2F). The behavioral difference was confirmed by EEG recordings. Energy spectra of representative epileptic EEGs recorded from Het::LGI1^WT^ and Het::LGI1^W183R^ mice are shown in Fig. 2G and the absolute power at each firing frequency is shown in Fig. 2H. These results indicated that expressing LGI1^W183R^ in excitatory neurons augmented PTZ-induced seizure severity compared to expressing LGI1^WT^ (Fig. 2G, H). Taken together, we conclude that LGI1^W183R^ in excitatory neurons is sufficient to cause epileptic seizures in mice.

### LGI1^W183R^ causes hyperexcitability and firing irregularity in hippocampal pyramidal neurons

**Fig.3:**
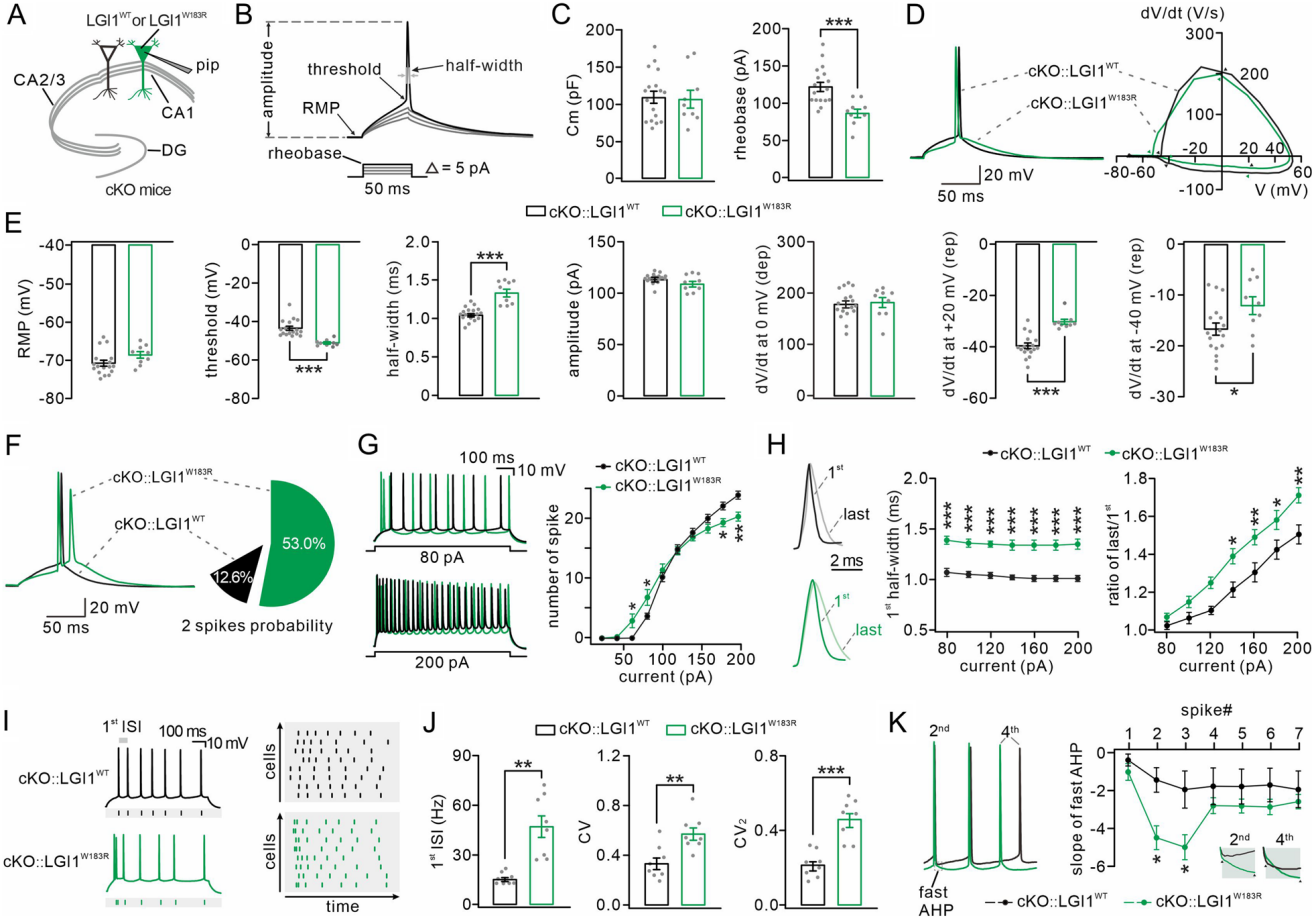
Hyperexcitability and spiking irregularity in hippocampal neurons expressing LGI1^W183R^.** A** Schematic of whole-cell recording in cKO CA1 pyramidal neurons expressing LGI1^WT^-GFP or LGI1^W183R^-GFP (pip: patch pipette). **B** AP evoked by a rheobase current (black), but not subthreshold currents (grey) (arrows, threshold, RMP, amplitude, and half-width. **C** Averages of membrane capacitance (Cm) and rheobase. **D** Left: example APs in cKO::LGI1^WT^ and cKO::LGI1^W183R^ neurons. Right: phase-plane plots for APs. The arrowheads show the measurement of threshold and dV/dt at 0, + 20 (repolarization) and − 40 mV. **E** Averages of RMP, threshold, half-width, amplitude, dV/dt at 0 mV, dV/dt at + 20 mV, and dV/dt at − 40 mV. **F** Left: example APs induced by rheobase current in cKO::LGI1^WT^ and cKO::LGI1^W183R^ neurons. Right: probabilities of doublet APs. **G** Left: example spikes recorded in cKO::LGI1^WT^ and cKO::LGI1^W183R^ neurons responding to 80-pA and 200-pA currents. Right: numbers of spikes as a function of injected currents. **H** Left: representative 1st and last spikes induced by 200-pA current. Middle: half-widths of 1st spikes induced by different currents plotted against to currents. Right: ratios of last *vs* 1st spikes were plotted against corresponding currents. **I** Left: example firing showing spike-timing reliability. Right: plots of intraburst jitters as a function of recording time. **J** Averages of 1^st^ ISI frequency, CV, and CV_2_. **K** Left: example AHPs. Right: plots of fast AHP as a function of spikes. Insets amplification of 2^nd^ and 4^th^ AHPs. See Additional file 8: Table S3 for statistics. Grey dots indicate individual data points. *P < 0.05. ** P < 0.01. *** P < 0.001

To investigate the mechanism by which LGI1^W183R^ regulate neuronal activity, we performed whole-cell recordings in hippocampal CA1 pyramidal neurons of cKO::LGI1^WT^ and cKO::LGI1^W183R^ mice aged P17–20 (Fig. 3A). Single action potentials (APs) were induced by rheobase current injection and their major kinetic parameters were analyzed (Fig. 3B). We found that cKO::LGI1^W183R^ neurons required a smaller rheobase than cKO::LGI1^WT^ neurons, but showed no difference in membrane capacitance between two groups (Fig. 3C). The plots of rate of change membrane potential (dV/dt) *vs* membrane potential revealed that AP waveform differed between cKO::LGI1^WT^ and cKO::LGI1^W183R^ neurons (Fig. 3D). In cKO::LGI1^W183R^ neurons, AP threshold was more hyperpolarized, the half-width was increased, and the values of dV/dt at + 20 mV and − 40 mV were increased (Fig. 3E). Meanwhile, AP amplitude and resting membrane potential (RMP) were unaltered (Fig. 3E). The altered AP parameters indicated that exogenous LGI1^W183R^ may influence the generation of AP. In fact, rheobase current that induced a single AP in cKO::LGI1^WT^ neurons could evoke doublet APs in cKO::LGI1^W183R^ neurons (Fig. 3F), suggesting that cKO::LGI1^W183R^ neurons are more excitable than cKO::LGI1^WT^ neurons.

Next, the population firings in pyramidal neurons were induced by a depolarizing step current. We found that an 80-pA current produced more spikes in cKO::LGI1^W183R^ neurons than in cKO::LGI1^WT^ neurons (Fig. 3G), suggesting that LGI1^W183R^ increases the firing potential. Unexpectedly, further study demonstrated that a 200-pA current produced fewer spikes in cKO::LGI1^W183R^ neurons, which was manifested by the input–output curves (injected current-number of spikes) obtained from cKO::LGI1^WT^ and cKO::LGI1^W183R^ neurons (Fig. 3G). Previous work has suggested that altered AP half-width is the reason for such bi-directional changes accompanying increasing stimulation intensities [36]. Indeed, we showed that AP half-width was increased by LGI1^W183R^ (Fig. 3E). To test if this is the case for population firing, we measured the half-width of the 1^st^ spike evoked by increasing stimuli (80–200 pA), and found that the values were always larger in cKO::LGI1^W183R^ neurons than in cKO::LGI1^WT^ neurons (Fig. 3H). Furthermore, we calculated the ratio of half-width of the last *vs* the 1^st^ spikes. Our results demonstrated that: the spike became wider in both cKO::LGI1^WT^ and cKO::LGI1^W183R^ neurons as time passed; but this ratio was always greater in cKO::LGI1^W183R^ neurons than in cKO::LGI1^WT^ neurons (Fig. 3H), which explained the reduced number of spikes upon stimulation with large currents.

Also, we noted that the 1^st^ interspike interval (ISI) in a firing was shorter in cKO::LGI1^W183R^ neurons (Fig. 3G). To clarify this point, we adjusted the intensity of injection currents (60–80 pA) to induce exactly 7 spikes in recorded neurons (Fig. 3I) [49], and measured two parameters: 1^st^ ISI frequency, which was augmented significantly in cKO::LGI1^W183R^ neurons (Fig. 3J), and the regularity of firing, which was characterized by the coefficient of variation of all ISIs (CV) and the coefficient of two consecutive ISIs (CV_2_) [50]. As shown by event rasters (Fig. 3I) and statistics of CV and CV_2_ (Fig. 3J), the firing became more irregular in cKO::LGI1^W183R^ neurons than in cKO::LGI1^WT^ neurons. It has been suggested that the ISI depends on the after-hyperpolarization potential (AHP) [34, 35], which can be separated into two parts, fast and slow AHPs [51, 52]. In our hands, we found that the fast AHP was decreased for the second and third spikes in cKO::LGI1^W183R^ neurons, but the difference gradually declined over time (Fig. 3K). Thus, these altered fast AHP may explain the irregularity of spontaneous spikes in cKO::LGI1^W183R^ neurons.

### K_v_1.1 activity is down-regulated in cKO::LGI1^W183R^ neurons

**Fig.4:**
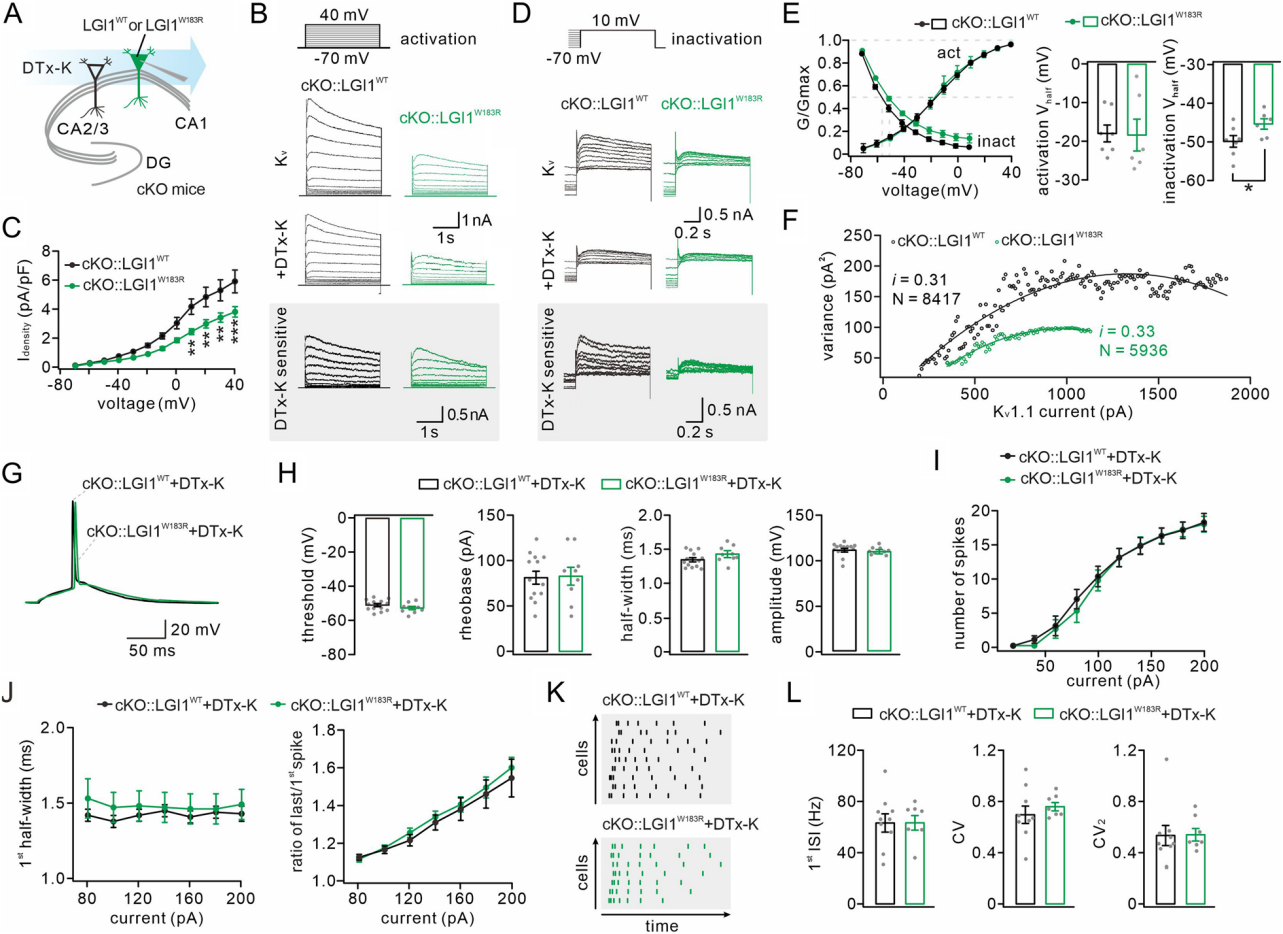
Downregulation of K_v_1.1 activity in cKO::LGI1^W183R^ neurons.** A** Schematic of whole-cell recording in cKO::LGI1^WT^ and cKO::LGI1^W183R^ neurons perfused with DTx-K. **B** Activated K^+^ current by stepped voltage pulses (− 70 to + 40 mV) in neurons before and after application of DTx-K (100 nM). K_v_1.1 current (DTx-K-sensitive) are from current subtraction. **C** K_v_1.1 current during the activation phase normalized to cell capacitance (current density) and plotted against command voltage. **D** The inactivation of K_v_1.1 current by stepped voltage pulses (− 70 to + 10 mV). **E** Left, steady-state activation and inactivation curves of K_v_1.1 current normalized to maximal conductance. Right, averages of half-voltages for the activation and inactivation curves. **F** Non-stationary noise analysis of K_v_1.1 activation at a command voltage of + 40 mV. Current variance is plotted against the amplitude at a given time point. Single-channel conductance and number of active channels are determined by fitting a parabola to the data points. **G** Example APs induced by rheobase in LGI1^WT^ and LGI1^W183R^ neurons treated with DTx-K. **H** Averages of AP threshold, rheobase, half-width, and amplitude with the addition of DTx-K. **I** Curves of spikes *vs* injected current in neurons perfused with DTx-K. **J** Left: averages of half-width of 1st spike induced by different current injections in neurons perfused with DTx-K. Right: ratios of last *vs* 1st spikes plotted against injected current. **K** Plots of intraburst jitters as a function of recording time in neurons perfused with DTx-K. **L** Average values of 1st ISI frequency, CV, and CV_2_ in neurons perfused with DTx-K. See Additional file 5: Tables S4 for statistics. Grey dots indicate individual data points. *P < 0.05. **P < 0.01. ***P < 0.001

A putative function of LGI1 is its modulation of glutamatergic transmission through binding ADAMs [24, 25]. However, the LGI1^W183R^ mutation unlikely plays this role, since it yields a secretion-defective LGI1 protein. Indeed, we found that neither the amplitude nor the frequency of mEPSCs was altered by LGI1^W183R^ expression in cKO neurons (Additional file 2: Fig. S2). Alternatively, LGI1^W183R^ may act on ion channels, as we had previously demonstrated that K_v_1 is down-regulated in cortical neurons upon LGI1 ablation [18]. To test this possibility, we made whole-cell recordings in cKO hippocampal neurons expressing LGI1^WT^-GFP or LGI1^W183R^-GFP with perfusion of DTx-K (a specific K_v_1.1 antagonist [53, 54]) (Fig. 4A). By applying a series of stepped voltage pulses to neurons, we obtained K_v_1.1 current by subtracting DTx-K-sensitive current from overall K^+^ current (Fig. 4B). Our results showed an overall decrease in K_v_1.1 current in cKO::LGI1^W183R^ neurons (Fig. 4B), indicating that LGI1^W183R^ inhibits K_v_1.1 current. This conclusion was strengthened by analyzing the activation and inactivation of K_v_1.1 current, which were defined as the currents evoked by depolarizing voltages and the currents evoked by 3-s inactivating pre-pulses, respectively [18]. As shown by normalized conductance recorded at stepped voltages (from − 70 to + 40 mV), both the activation and the inactivation of K_v_1.1 current were reduced by LGI1^W183R^ expression (Fig. 4C, D). Further kinetics analysis showed no effect of LGI1^W183R^ on the slope of activation curve and half-activation voltage, and that there was an increase in half-inactivation voltage, but not the slope of the inactivation curve, following LGI1^W183R^ expression (Fig. 4E). These analyses reveal that LGI1^W183R^ exerts strong regulatory effects on K_v_1.1 activity, which can alter the waveform of the AP and spiking pattern [36, 55–58]. To determine the cause of K_v_1.1 current reduction, i.e. whether it is due to a reduction of single-channel conductance or the number of active channels, we performed non-stationary noise analysis on the activation of K_v_1.1 in cKO neurons expressing LGI1^WT^ or LGI1^W183R^ at a command voltage of + 40 mV [59, 60]. Plotting current variance as a function of current amplitude yielded a parabola, whose parameters are suitable to determine single-channel conductance and the number of active channels [59, 60]. Our analysis indicated that LGI1^W183R^ expression reduced the number of active K_v_1.1 channel (8417 for LGI1^WT^ and 5936 for LGI1^W183R^), while single-channel conductance was not affected (0.31 for LGI1^WT^ and 0.33 for LGI1^W183R^) (Fig. 4F). These data show that the reduction in K_v_1.1 current is due to a reduced number of active channels.

If LGI1^W183R^ reduces K_v_1.1 activity, it is reasonable to assume that inhibiting K_v_1.1 is able to annihilate the difference in intrinsic excitability between cKO::LGI1^WT^ and cKO::LGI1^W183R^ neurons. To test this idea, we perfused DTx-K onto cKO neurons expressing either LGI1^WT^ or LGI1^W183R^, and examined the AP and spikes. Under this condition, AP waveform showed similar kinetics in cKO::LGI1^WT^ and cKO::LGI1^W183R^ neurons (Fig. 4G). The statistics showed no difference between cKO::LGI1^WT^ and cKO::LGI1^W183R^ neurons in a number of AP parameters, including threshold, rheobase, half-width, and peak amplitude (Fig. 4H). Again with stepped current injection, we compared the number of spikes, half-width of 1^st^ spike, half-width ratio of the last *vs* 1^st^ spike, and spiking regularity, which were shown to differ between cKO::LGI1^WT^ and cKO::LGI1^W183R^ neurons (Fig. 3). With the perfusion of DTx-K, no difference was found in the numbers of spikes for all intensities of injected currents, as shown by the input–output curves (Fig. 4I). We induced 7 spikes in cKO::LGI1^WT^ and cKO::LGI1^W183R^ neurons with the perfusion of DTx-K. Likewise, DTx-K eliminated the differences in the half-width of 1st spike and half-width ratio of the last *vs* 1st spike, when cKO::LGI1^WT^ and cKO::LGI1^W183R^ neurons were injected with the same current (Fig. 4J). In addition, DTx-K application changed the firing of cKO::LGI1^WT^ neurons, making the pattern equal to that of cKO::LGI1^W183R^ neurons (Fig. 4K). The statistics showed that the values of 1^st^ ISI frequency, CV, and CV_2_ were all increased by DTx-K application in cKO::LGI1^WT^ neurons, while these parameters were not altered in cKO::LGI1^W183R^ neurons (Fig. 4L), thereby eliminating the differences between two groups. Taken together, we conclude that a reduction in K_v_1.1 activity is the cause of the abnormal excitability in cKO neurons expressing LGI1^W183R^.

### K_v_1.1 control spiking pattern of pyramidal neurons: evidence from computer simulation

**Fig.5:**
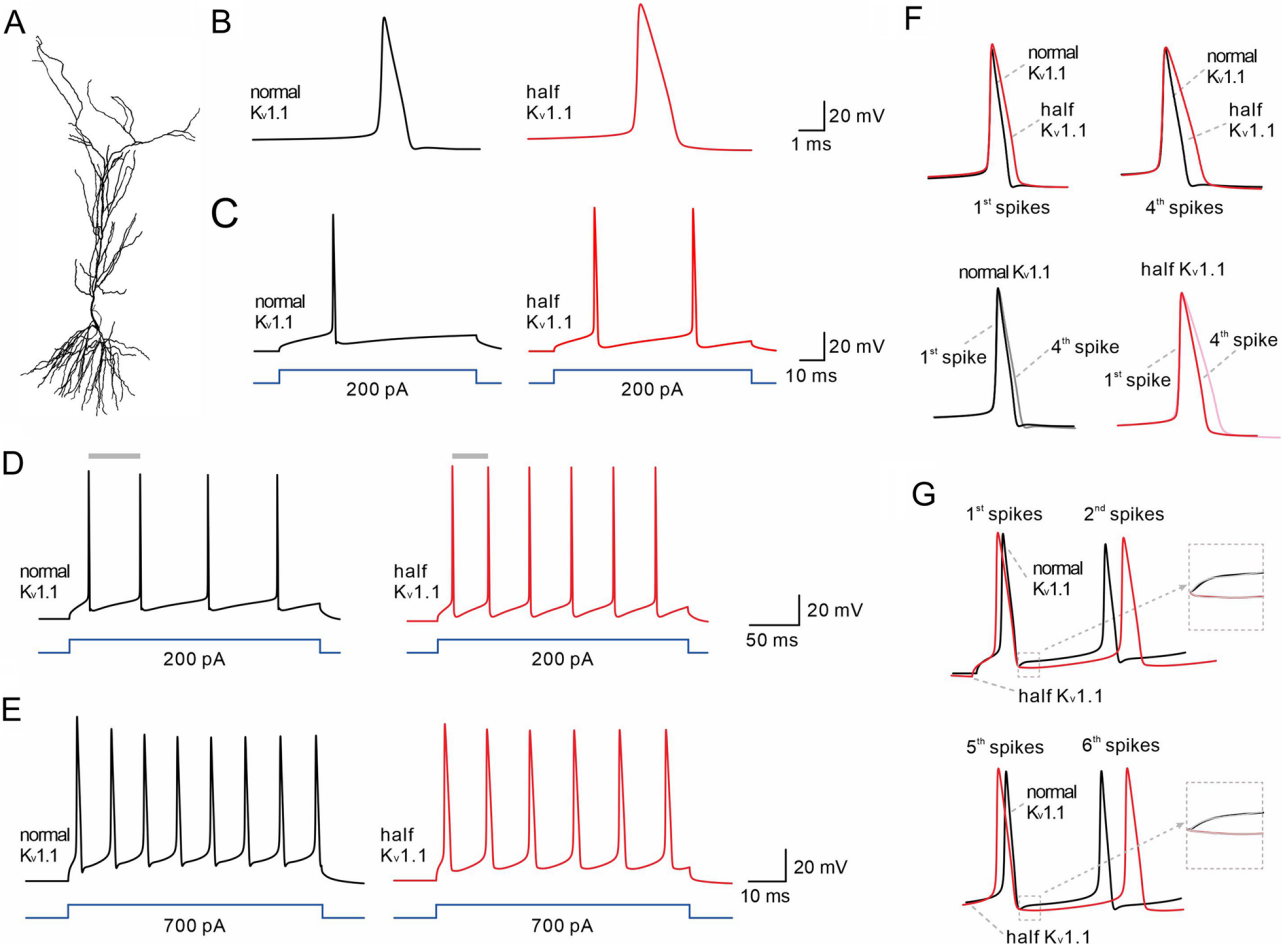
Computer model of CA1 neuronal firing pattern. **A** A schematic model of a CA1 pyramidal neuron. **B** APs evoked by rheobase in the cell model containing normal (0.02) or the half (0.01) of K_v_1.1. **C** The same strength of stimulation induces a single AP with K_v_1.1 (0.02), but doublet APs with insufficient K_v_1.1 (0.01). **D** A reduction in K_v_1.1 results in more firing when the cell receives a 200-pA current injection (400 ms) (gray bars, intervals between 1st and 2nd spikes). **E** Reduced K_v_1.1 results in less firing when the cell receives a 700-pA current injection (400 ms). **F** The amplifications of 1^st^ and 4^th^ spikes induced by 200-pA current. Note the difference in the half-width between 1^st^ and 4^th^ spikes, or between normal and half K_v_1.1 conditions. **G** The amplifications of 1^st^ and 5^th^ AHPs and fitting analysis (gray lines) of AHP currents with normal or half K_v_1.1

To better elucidate the contribution of K_v_1.1 to neuronal firing, we constructed a neuronal model containing a repertoire of voltage-dependent ion channels (Fig. 5A) [43, 61–63]. The conductance densities were adjusted to generate an AP at a threshold of − 20 mV above RMP, and firing was elicited at suprathreshold currents.

K_v_1.1 density was set at half of normal to mimic the reduction of K_v_1.1 activity caused by LGI1^W183R^. Our simulation showed that K_v_1.1 reduction significantly widened the AP (Fig. 5B). We then applied a rheobase current, which was sufficient to evoke a single AP in a cell containing normal K_v_1.1, to a cell containing half of the K_v_1.1. We found that the rheobase current induced a single AP in the cell with normal K_v_1.1, but induced doublet APs in the cell with insufficient K_v_1.1 (Fig. 5C). These results confirm that the pattern of neuronal APs is dependent on K_v_1.1.

Next, we investigated the dependence of firing on the density of K_v_1.1. Two levels of current injections (200 pA and 700 pA) were applied to the cell model as the low and high intensities of current injection, respectively. With 400-ms duration, 200-pA current induced 4 APs with normal K_v_1.1, but 6 APs with half K_v_1.1 (Fig. 5D). Moreover, the ISI between first two spikes was reduced in the cell with half K_v_1.1 (Fig. 5D). However, 700-pA stimulation led to 8 spikes with normal K_v_1.1, but 6 spikes with reduced K_v_1.1 (Fig. 5E). The bidirectional change in the number of spikes with low and high intensities of stimulation is consistent with our whole-cell recording results. We amplified the spikes induced by 200-pA stimulation, and found that K_v_1.1 reduction increased the half-width of 1st and 4th spikes (Fig. 5F). Moreover, the half-width ratio of 4th/1st spikes appeared more significant with reduced K_v_1.1 (Fig. 5F), also consistent with our whole-cell recordings.

The resurgence of AHP current appeared slower with half K_v_1.1 (Fig. 5E), which may explain the altered firing pattern. To test this point, we analyzed AHPs in early and late spikes with normal or fewer K_v_1.1 channels. The polynomial fitting showed that K_v_1.1 reduction caused a different pattern of fast AHP between the 1st to and the 2nd spikes, that is, the AHP tended to depolarize with normal K_v_1.1 (slope coefficient: 68), but tended to hyperpolarize with reduced K_v_1.1 (slope coefficient: − 214) (Fig. 5G). Interestingly, in the follow-up AHPs, the difference began to decrease, showing that the slope coefficient was 30 for normal K_v_1.1 and − 3 for fewer Kv1.1 channels (Fig. 5G). Therefore, these data suggest that K_v_1.1 reduction permits different firing pattern by delaying the onset of AHP currents.

### Restoring K_v_1.1 alleviates seizure susceptibility in cKO::LGI1^W183R^ mice

**Fig.6:**
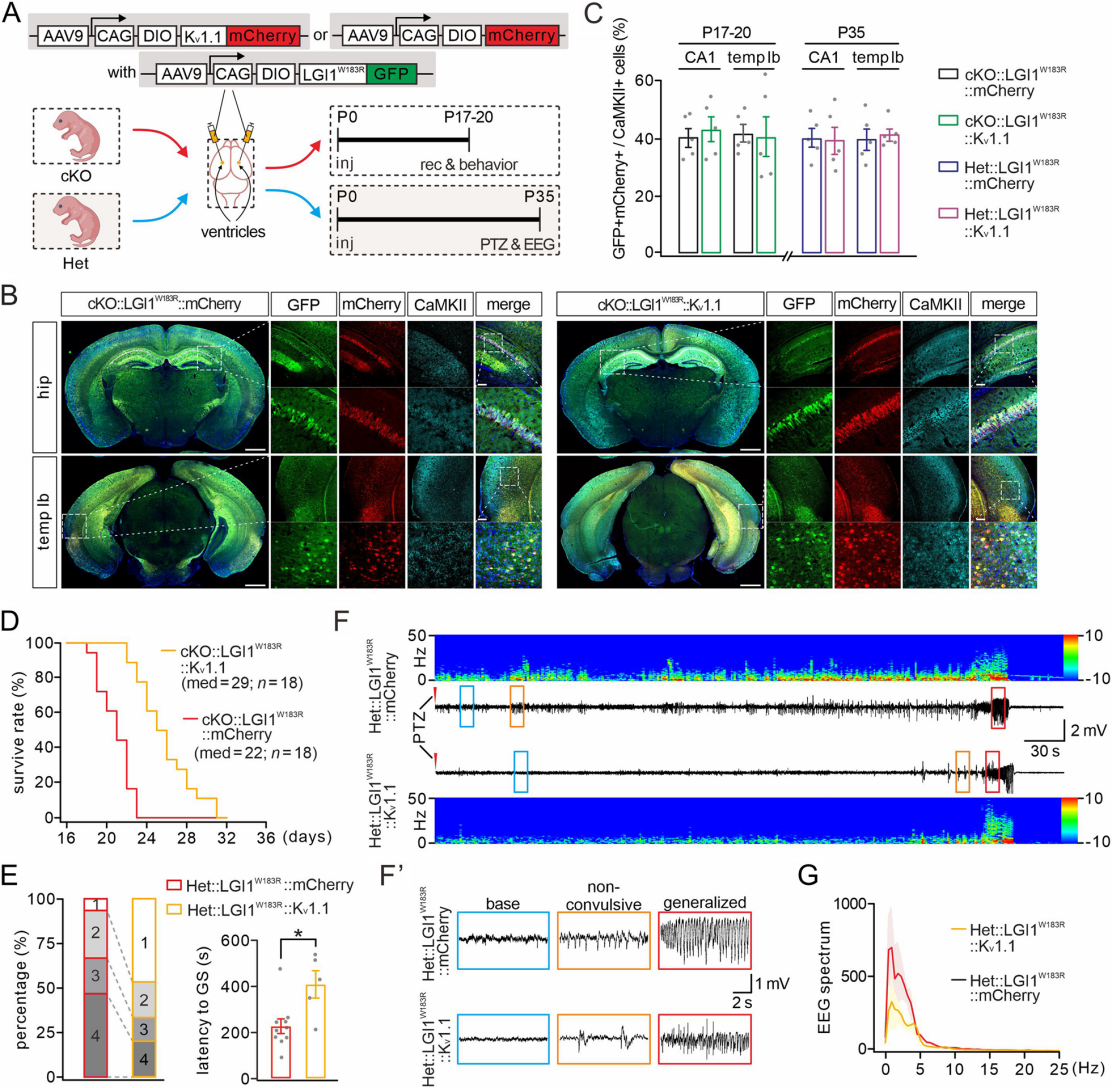
Reduced seizure susceptibility after Kv1.1 restoring. **A** AAV9-DIO-Kv1.1-mCherry or AAV9-DIO-mCherry accompanied by AAV9-DIO-LGI1^W183R^-GFP expressed in excitatory neurons. The viruses were injected bilaterally into the ventricles of cKO or Het mice at P0. **B** Representative images for quadruple-fluorescence (GFP, mCherry, CaMKII and DAPI) in the hippocampus (hip) and temporal cortex (temp lb) of cKO mice (P17). Scale bars: 1 mm (whole brain) and 50 μm (magnified). **C** The ratios of numbers of GFP + mCherry + *vs* CaMKII + cells (*n* = 5 mice per group) at P17-20 were 41 ± 3 (CA1; cKO::LGI1^W183R^::mCherry) and 44 ± 4 (CA1; cKO::LGI1^W183R^::K_v_1.1), *P* = 0.64; 42 ± 3 (temp lb; cKO::LGI1^W183R^::mCherry) and 41 ± 7 (temp lb; cKO::LGI1^W183R^::K_v_1.1), *P* = 0.88. At P35, the ratio were 41 ± 3 (CA1; cKO::LGI1^W183R^::mCherry) and 40 ± 5 (CA1; cKO::LGI1^W183R^::K_v_1.1), *P* = 0.92; 40 ± 4 (temp lb; cKO::LGI1^W183R^::mCherry) and 42 ± 2 (temp lb; cKO::LGI1^W183R^::K_v_1.1), *P* = 0.72. **D** Kaplan–Meier curves. **E** Reactions to PTZ injection of Het::LGI1^W183R^::mCherry and Het::LGI1^W183R^::K_v_1.1 mice. Latency to generalized seizure (GS): 228 ± 31 s (Het::LGI1^W183R^::mCherry; *n* = 10) and 409 ± 59 s (Het::LGI1^W183R^::K_v_1.1; *n* = 5), *P* = 0.033. **F** Example EEG recordings in cKO::LGI1^W183R^::mCherry and cKO::LGI1^W183R^::K_v_1.1 mice (P35) during PTZ-induced seizures. **F’** Enlarged view of EEGs in **F**. **G** Spectral analysis of EEGs. *P < 0.05

Having demonstrated that reduced activity of K_v_1.1 by LGI1^W183R^ expression is responsible for epileptogenesis, an interesting question was whether the seizures can be ameliorated and whether the lifespan can be prolonged by restoring K_v_1.1. To do so, we injected AAV9-DIO-LGI1^W183R^-GFP with AAV9-DIO-K_v_1.1-mCherry or AAV9-DIO-mCherry bilaterally into the ventricles of Het or cKO mice at P0 (Fig. 6A). Using this approach, LGI1^W183R^ and K_v_1.1, as exogenous proteins, were simultaneously expressed in excitatory neurons from cKO or Het mice. Later, the mice expressing LGI1^W183R^ and K_v_1.1-mCherry or mCherry were subjected to video monitoring of autonomous or PTZ-induced seizures and/or electrophysiological recordings (Fig. 6A). The expression of LGI1^W183R^ and K_v_1.1 was confirmed by the fluorescence of GFP and mCherry, respectively. Figure 6B shows that both LGI1^W183R^-GFP and K_v_1.1-mCherry signals were robustly present in the temporal cortex and the hippocampus of cKO mice. Meanwhile, the fluorescent signals of GFP and mCherry also co-localized with CaMKIIα-Cre expressing neurons (Fig. 6B). Counting the numbers of GFP + mCherry + and CaMKIIα + neurons showed no difference in the ratio of GFP + mCherry + neurons among CaMKIIα + neurons either between mCherry and K_v_1.1 groups at two developmental stages (Fig. 6C). These results indicate that exogenous LGI1^W183R^ and K_v_1.1 are robustly expressed in excitatory neurons. Again, GFP and mCherry signals did not co-localize with the marker proteins for other major types of nerve cells (Additional file 3: Fig. S3).

The majority of cKO::LGI1^W183R^::mCherry mice (15/18) died before P21 with a median lifetime of 22 days (Fig. 6D). In contrast, the majority of cKO::LGI1^W183R^::K_v_1.1 littermates survived beyond this period with a median lifetime of 29 days (Fig. 6D). Video monitoring showed that cKO::LGI1^W183R^::K_v_1.1 mice exhibited a significant reduction in seizures (Additional file 10: Movie S2 and Additional file 5: Table S2). Furthermore, we examined PTZ-induced seizures in Het::LGI1^W183R^::K_v_1.1 and Het::LGI1^W183R^::mCherry mice, and found that seizure severity was significantly less in Het::LGI1^W183R^::K_v_1.1 mice: most (10/15) Het::LGI1^W183R^::mCherry mice were at stages 3/4, whereas most Het::LGI1^W183R^::K_v_1.1 mice (10/15) were at stages 1/2 and had an increased latency to generalized seizures (Fig. 6E). Energy spectra of epileptic EEGs recorded from Het::LGI1^W183R^::mCherry and Het::LGI1^W183R^::K_v_1.1 mice are shown in Fig. 6F and the absolute power for at each firing frequency is shown in Fig. 6G. Our results indicated that restoring K_v_1.1 significantly reduced PTZ-induced seizure severity in Het::LGI1^W183R^ mice. In summary, we conclude that restoring K_v_1.1 alleviates seizure susceptibility and extends the lifespan of cKO::LGI1^W183R^ mice.

### Restoration of cKO::LGI1^W183R^ neuronal excitability by expressing K_v_1.1

**Fig.7:**
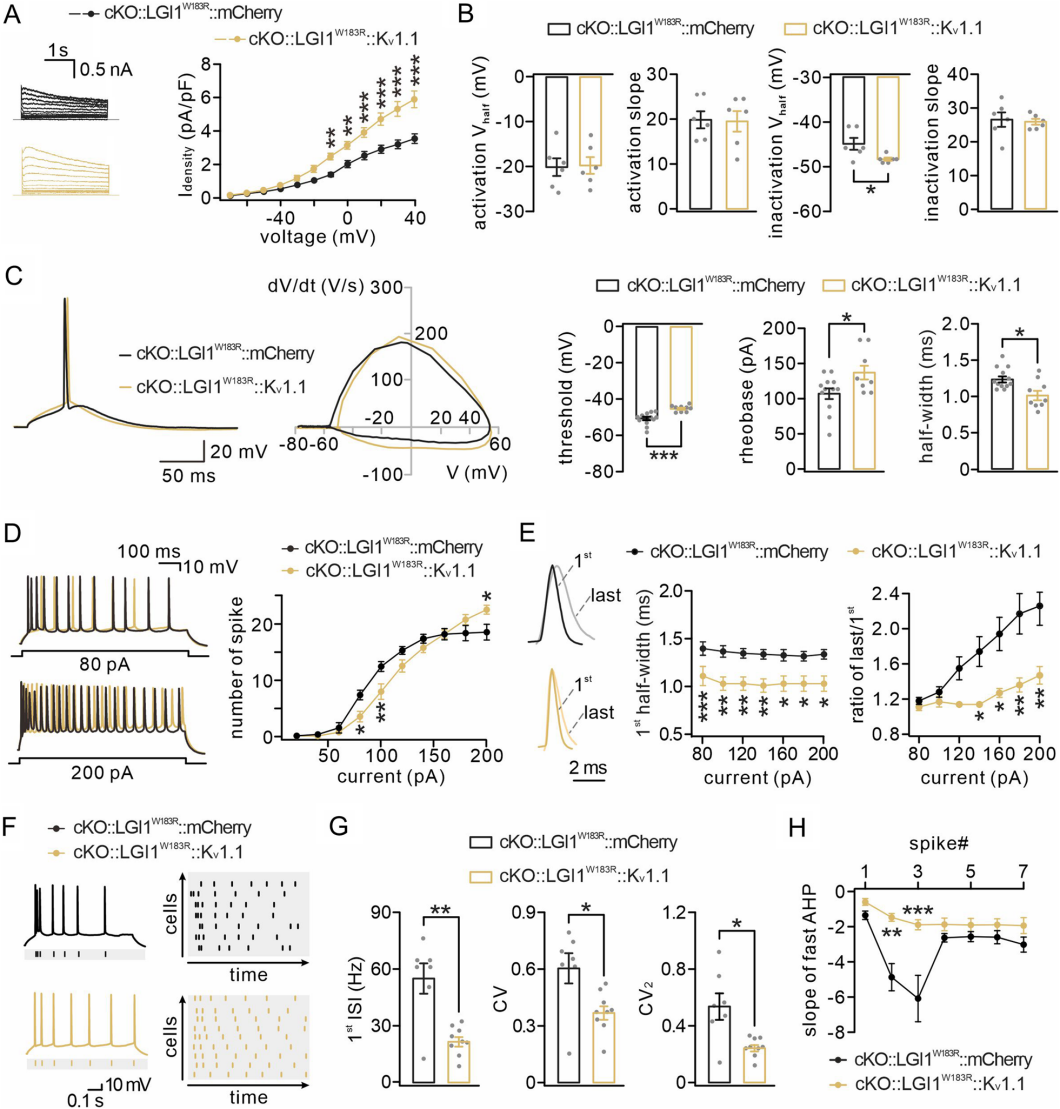
Restoring K_v_1.1 rescues neuronal excitability. **A** Left: example K_v_1.1 current activated by stepped pulses in cKO::LGI1^W183R^::mCherry and cKO::LGI1^W183R^::K_v_1.1 pyramidal neurons. Right: plots of current–voltage relationship of K_v_1.1 current. **B** Averages of activation half-voltage, slope of activation curve, inactivation half-voltage, and slope of the inactivation curve. **C** Left: example APs and phase-plane plots. Right: averages of threshold, rheobase, and half-width of APs. **D** Left: example spikes of cKO::LGI1^W183R^::mCherry and cKO::LGI1^W183R^::K_v_1.1 neurons. Right: input–output curves showing numbers of spikes as a function of injected current. **E** Left: example 1st and last spikes induced by a 200-pA current in cKO::LGI1^W183R^::mCherry and cKO::LGI1^W183R^::K_v_1.1 neurons. Middle: half-width of 1st spike induced by different currents plotted against current. Right: ratios of the last *vs* 1st spike plotted against inject current. **F** Left: example firing showing spike-timing reliability. Right: plots of intraburst jitter as a function of recording time. **G** Averages of 1st ISI frequency, CV, and CV_2_. **H** Plots of fast AHP as a function of spikes. See Additional file 7: Table S5 for statistics. Grey dots indicate individual data points. *P < 0.05. **P < 0.01. ***P < 0.001

If restoring K_v_1.1 reduces epileptic seizures, it should be able to reverse the impaired intrinsic excitability in neurons expressing LGI1^W183R^. To address this point, we expressed K_v_1.1 in excitatory neurons of cKO::LGI1^W183R^ mice and made whole-cell recordings from these neurons. We found that expressing K_v_1.1, but not control mCherry, effectively restored K_v_1.1 current in cKO::LGI1^W183R^ neurons (Fig. 7A). Kinetics analysis showed that expressing K_v_1.1 rescued the defective activation and inactivation of K_v_1.1 currents, showing a decreased half-inactivation voltage in cKO::LGI1^W183R^ neurons expressing K_v_1.1 (Fig. 7B).

We continued to study the effects of K_v_1.1 expression on the AP, firing frequency and regularity. First, expressing K_v_1.1 made the AP waveform of cKO::LGI1^W183R^ neurons similar to cKO::LGI1^WT^ neurons, and significantly restored the threshold, rheobase, and half-width that were changed by LGI1^W183R^ (Fig. 7C). Second, cKO::LGI1^W183R^::K_v_1.1 neurons had fewer spikes in response to current injection (Fig. 7D). Third, the half-width of spikes was significantly reduced in cKO::LGI1^W183R^::K_v_1.1 neurons in response to stepped current injections (Fig. 7E). Moreover, the half-width ratio of last *vs* 1^st^ spikes was also significantly reduced by K_v_1.1 expression for the high intensity currents (Fig. 7E), which explains the increased number of spikes under these conditions (Fig. 7D). Fourth, the spiking irregularity of cKO::LGI1^W183R^ was rescued by the expression of K_v_1.1. As shown by 7 spikes and activity raster (Fig. 7F), K_v_1.1 expression relaxed the firing pattern of cKO::LGI1 ^W183R^ neurons, making it close to that of WT neurons. The statistics indicated that 1^st^ ISI, CV, and CV_2_ all decreased in cKO::LGI1^W183R^::K_v_1.1 neurons in comparison with cKO::LGI1^W183R^::mCherry neurons (Fig. 7G). Finally, we measured the fast AHPs of spikes in cKO::LGI1^W183R^::mCherry and cKO::LGI1^W183R^::K_v_1.1 neurons, and found that K_v_1.1 rescued the deficit of the fast AHP in cKO::LGI1^W183R^ neurons (Fig. 7H). This result explains the return of spiking regularity in cKO::LGI1^W183R^::K_v_1.1 neurons. Taken together, restoring K_v_1.1 rescues the impaired AP kinetics and firing pattern in cKO::LGI1^W183R^ neurons.

## Discussion

Endogenous LGI1 has two homologous isomers: the shorter is secreted, while the longer is not secreted and is retained inside cells [11], suggesting that LGI1 may affect neuronal activity through different mechanisms. Secreted LGI1 acts as a ligand to bind to ADAM22 at postsynaptic sites of excitatory synapses and regulates synaptic development and AMPA receptor-mediated neurotransmission [24]. If LGI1 is knocked out globally, synaptic transmission is disrupted and neuronal excitability is altered, resulting in autonomous epilepsy and pre-mature death in mice [28, 64]. In contrast, no epileptic symptoms are observed when LGI1 is knocked out in either interneurons or astrocytes [19]. These findings demonstrate the crucial roles of LGI1 secreted from excitatory neurons in epileptogenesis.

These results may lead to a mystery: why can’t the LGI1 secreted from other types of nerve cell compensate for the loss of LGI1 from excitatory neurons? As a matter of fact, it has been shown that LGI1 is expressed in PV-positive interneurons, astrocytes and oligodendrocytes [19, 20]. In addition, LGI1 can act on adjacent synapses through both paracrine and autocrine mechanisms [25]. These results imply that the LGI1 from interneurons and astrocytes would act on excitatory synapses, which may make the LGI1 from excitatory neurons appear dispensable. Our previous work solved this paradox, showing that LGI1 deficiency can disable K_v_1 channels in excitatory neurons, thereby increasing neuronal firing [18]. Thus, functional LGI1 inside neurons may also be critical for maintaining normal neuronal function.

Compared to mouse models with LGI1 deficiency, the situation of dysfunctional LGI1 is more complicated in ADLTE patients. Among 41 LGI1 missense mutations found in familial ADLTE patients, more than half are secretion-defective with different secretion probabilities [3–12, 26, 28]. While the ADLTE patients with secretion-defective LGI1 mutations display epileptic seizures as well, the role of secretion-defective LGI1 on neuronal activity has been unclear. Yokoi et al. [28]*.* examined a mouse model of familial epilepsy with the secretion-defective LGI1 mutation, LGI1^E383A^, and their findings suggested that this mutation may damage the structure of LGI1 protein and cause its rapid degradation, resulting in a condition similar to the deletion of LGI1. Moreover, they demonstrated that an improvement of LGI1 secretion by 4PBA, a chemical corrector, reduces the risk of developing epilepsy in mouse with LGI1 secretion-defective mutations [28]. However, there is a lack of evidence for the assertion that secretion-defective LGI1 mutations lead to the degradation of LGI1 protein. In fact, the LGI1^E383A^ mutation occurs at the EPTP domain of LGI1 [26, 28], which may only affect the binding between LGI1 and ADAM22 [26]. Thus, it is unclear whether and what role the secretion-defective LGI1 proteins play in the cytosol.

Here, we showed that with LGI1^W183R^, a novel secretion-defective mutation, LGI1 protein is robustly expressed and its ubiquitination level is not changed by the mutation. Therefore, the secretion-defective mutations of LGI1 may affect neuronal activity through an alternative mechanism, not only degradation. It has been shown that LGI1 co-localizes with K_v_1 at the axonal initial segment [22] and LGI1 regulates the inactivation of K_v_1.1 channel [21]. These studies suggest that LGI1 protein in the cytosol is critical to the stability and regulates the activity of K_v_1 channels. Indeed, we here provide evidence showing that LGI1^W183R^ mutation negatively regulates K_v_1.1 activity and leads to neuronal hyperactivity, resulting in epileptic seizures. Therefore, our findings answer how neuronal hyperactivity is induced by a secretion-defective LGI1 mutation and this intrinsic disorder is not saved by paracrine LGI1. Based on existing studies, we speculate that LGI1 acts on neuronal activity by regulating the function of excitatory synapses and the activity of K_v_1 channels. Recently, Baudin et al*.* [23] found that inhibiting K_v_1.1 alters neuronal excitability, mimicking anti-LGI1-associated seizures and supporting our findings. We found that dysfunctional K_v_1.1 increases neuronal firing irregularity, consistent with the results reported in cortical neurons [49]. Furthermore, the irregular discharges of hippocampal neurons concur with the emergence of seizure in epileptic rats [65]. Based on these studies, we conclude that irregular spikes caused by K_v_1.1 dysregulation are partly responsible for the epilepsy in LGI1^W183R^ mice. Yet, LGI1 may have other functional partners, which may explain why restoring K_v_1.1 does not fully rescue the life span of LGI1^W183R^ mice. In addition to the molecular partners of LGI1 in excitatory neurons, we cannot neglect the potential roles of LGI1 in inhibitory neurons and non-neuronal complexes, such as astrocytes and oligodendrocytes, since LGI1 also occurs in inhibitory synaptic sites as well as neuronal-glial protein complex [20]. Another intriguing question is that LGI1 may play roles in glioma genesis and oligodendrocyte differentiation and myelination [14, 66].

LGI1 mutations are distributed in all domains of LGI1, implying the distinct actions of these mutations. During the treatment of ADLTE patients carrying a certain LGI1 missense mutation, it is necessary to clarify the specific function of the mutation and determine its major action. After all, > 40 LGI1 mutations must be linked to distinct secretion probabilities and regulatory actions. In this way, it is essential for precision medicine to conduct large-scale functional analysis of human familial ADLTE-linked mutations. At present, other three mutations, c. 535t > c, c.598t > c, and c.641t > c, have been found in the C-cap domain as well. It is known that these mutations are also secretion-defective [26, 28], but unknown whether they affect K_v_1 channel activity. In fact, the binding affinity to partner molecules has been studied for almost all no mutations, except for the binding between a few mutations of LGI1 and ADAM22 [26]. Therefore, it is necessary to clarify the impact of LGI1 mutations on the structure and binding ability to its partner molecules in the future.

## Conclusions

In sum, we found a novel pathogenic variant of LGI1 in a Chinese family suffering ADLTE, expanding the spectrum of causative variants of LGI1. We unveiled the pathogenic mechanism exhibited by the p.Trp183Arg missense mutation in epileptic seizures, showing that this mutation produced secretion-defective LGI1^W183R^ protein, which caused the hyperexcitability and firing irregularity of excitatory neurons, and epileptic seizures in mice by downregulating K_v_1.1 activity. Moreover, restoring K_v_1.1 in excitatory neurons was able to correct the deficits in firing and ameliorate seizure susceptibility. Therefore, our work reveals a new mechanism by which a secretion-defective LGI1 protein causes neuronal dysfunction and familial epilepsy.

## Supplementary Information

**Additional file 1.** Figure S1. Expressing LGI1W183R in excitatory neurons does not affect other types of nerve cells. AAV9-DIO-LGI1W183R-GFP was injected bilaterally into the ventricles of cKO mice (P0). Representative images for triple fluorescence of GFP, individual marker proteins (PV, GFAP, Iba1, and NeuN), and DAPI, show that LGI1W183R is not expressed in PV-positive interneurons, astroglia (GFAP) and microglia (Iba1) in the hippocampus and temporal cortex of cKO mice (P17). Scale bars: 1 mm (whole brain) and 50 μm (magnified).

**Additional file 2.** Figure S2. Unchanged excitatory transmission in LGI1W183R neurons. (A) Example mEPSCs from cKO::LGI1WT and cKO::LGI1W183R mice (P17). (B) Cumulative plots of mEPSC amplitude. (C) Mean values of mEPSC frequency: 0.23 ± 0.02 Hz (cKO::LGI1WT; n = 7) and 0.22 ± 0.03 Hz (cKO::LGI1W183R ; n = 9), P = 0.88.

**Additional file 3.** Figure S3. Restoring Kv1.1 in excitatory neurons does not affect other types of nerve cells. AAV9-DIO-LGI1W183R-GFP and AAV9-DIO-Kv1.1-mCherry were bilaterally injected into the ventricles of cKO mice (P0). Representative images for quadruple fluorescence of GFP, mCherry, individual marker proteins (PV, GFAP, Iba1, and NeuN), and DAPI, show that LGI1W183R is not expressed in PV-positive interneurons, astroglia (GFAP) and microglia (Iba1) in the hippocampus and temporal cortex of cKO mice (P17). Scale bars: 1 mm (whole brain) and 50 μm (magnified).

**Additional file 4. Table S1.** The statistics for Fig. 1G, 1H, 1J, and 1K.

**Additional file 5. Table S2.** The statistics of spontaneous seizures.

**Additional file 6. Table S3.** The statistics for Fig. 3C, 3G, 3H, 3J and 3K.

**Additional file 7. Table S4.** The statistics for Fig. 4C, 4D, 4H, 4I, 4J and 4L.

**Additional file 8. Table S5.** The statistics for Fig. 7A, 7B, 7C, 7D, 7E, 7G and 7H. 

**Additional file 9.** Movie 1. A cKO::LGI1WT mouse (P20; right) behaves normally, while another cKO::LGI1W183R mouse (P20; left) displays epileptic seizures. MPEG-4 format, 4.8 MB.

**Additional file 10.** Movie 2. A cKO::LGI1W183R::mCherry mouse (P20; right) displays epileptic seizures, while another cKO::LGI1W183R::Kv1.1 mouse (P20; left) behaves normally. MPEG-4 format, 6.2 MB

**Additional file 11. ** Proband clinical information. 

**Additional file 12. ** Proband family sequencing result. 

## Data Availability

Any additional data and materials are available from corresponding authors on reasonable request.

## Abbreviations

ADAMs: A distintegrin and metalloproteinases
ADLTE: Autosomal dominant lateral temporal epilepsy
AHP: After-hyperpolarization potential
AP: Action potential
CHX: Cycloheximide
C_m_: Membrane capacitance
C_V_: Coefficient of variation
EEG: Electroencephalogram
GS: Generalized seizure
ISI: Interspike interval
LGI1: Leucine-rich glioma inactivated 1
MRI: Magnetic resonance imaging
PTZ: Pentylenetetrazole
PV: Parvalbumin
RMP: Resting membrane potential
