# Supplementary figures and images for: A patient-derived mutation of epilepsy-linked LGI1 increases seizure susceptibility through regulating Kv1.1
Source: Cell Biosci. 2023 Feb 20;13:34. doi: 10.1186/s13578-023-00983-y (PMC9940402; doi:10.1186/s13578-023-00983-y)

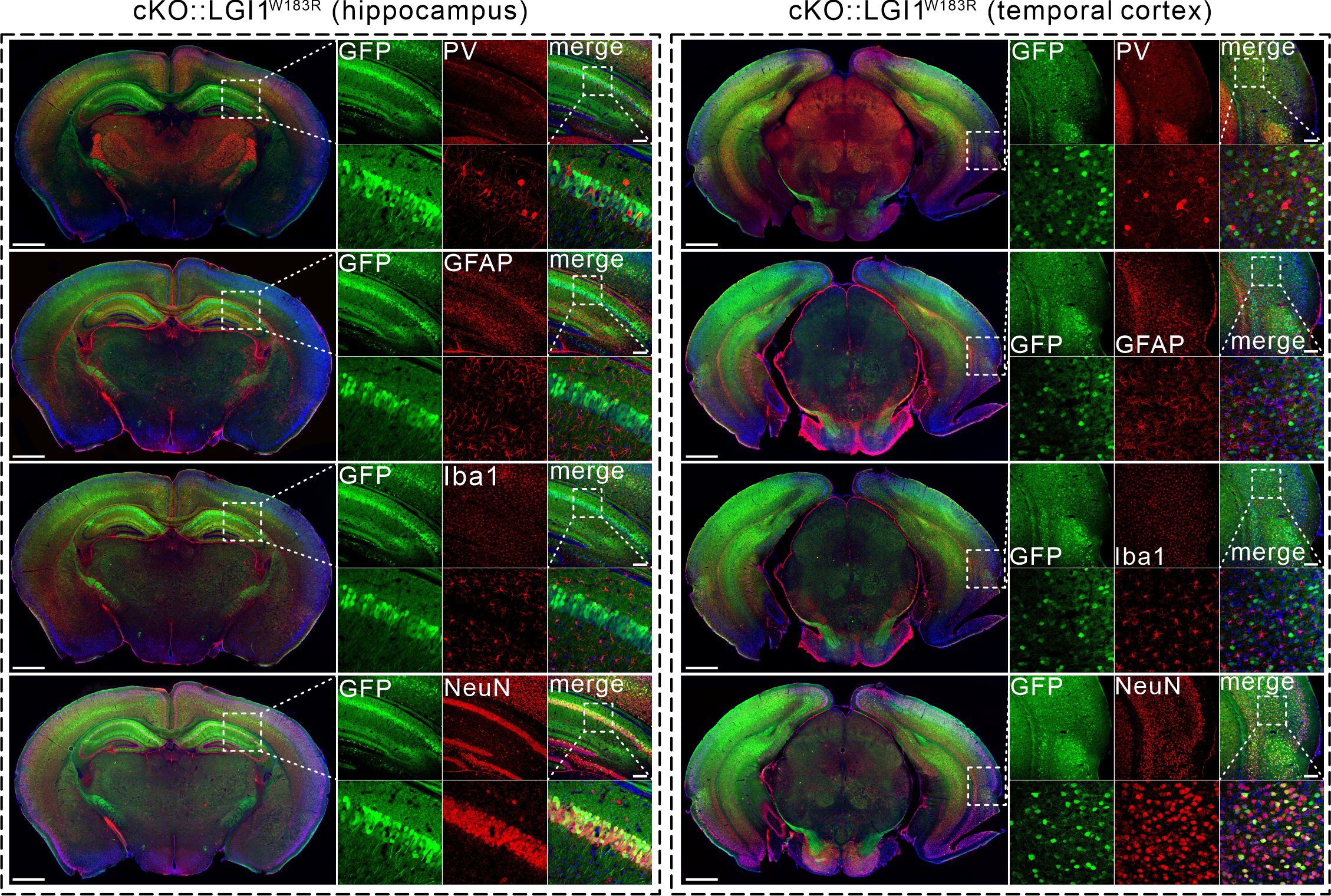

Supplement: Supplementary file 1 — Additional file 1. Figure S1. Expressing LGI1W183R in excitatory neurons does not affect other types of nerve cells. AAV9-DIO-LGI1W183R-GFP was injected bilaterally into the ventricles of cKO mice (P0). Representative images for triple fluorescence of GFP, individual marker proteins (PV, GFAP, Iba1, and NeuN), and DAPI, show that LGI1W183R is not expressed in PV-positive interneurons, astroglia (GFAP) and microglia (Iba1) in the hippocampus and temporal cortex of cKO mice (P17). Scale bars: 1 mm (whole brain) and 50 μm (magnified). [file 13578_2023_983_MOESM1_ESM.jpg]

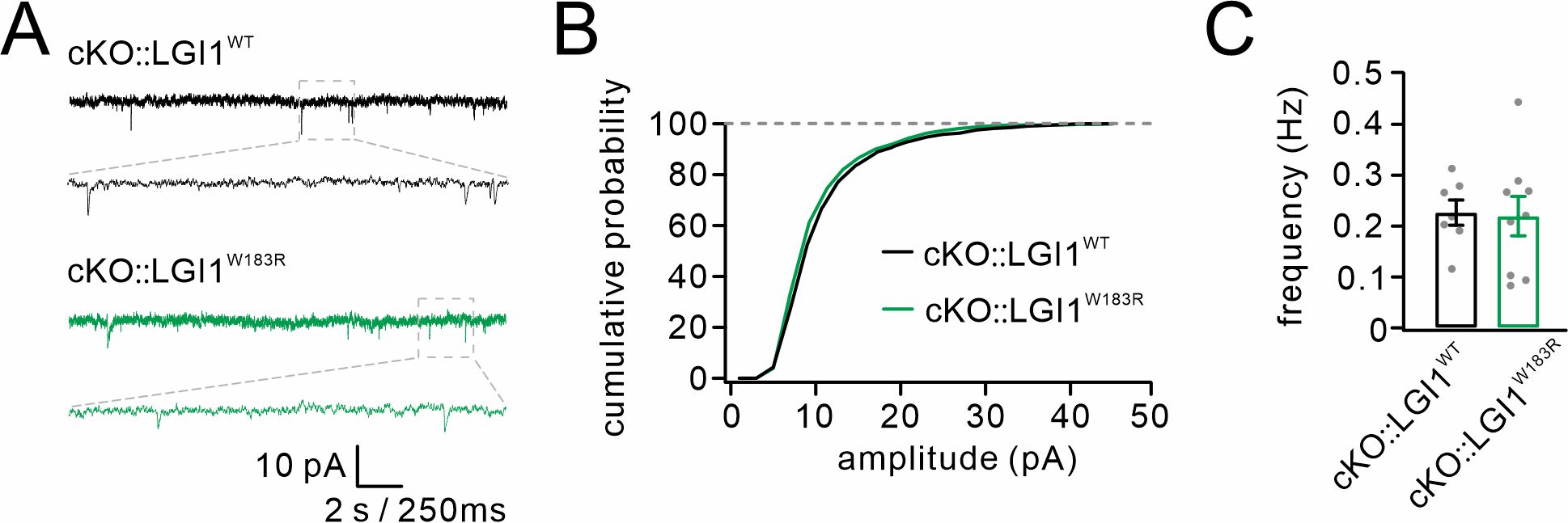

Supplement: Supplementary file 2 — Additional file 2. Figure S2. Unchanged excitatory transmission in LGI1W183R neurons. (A) Example mEPSCs from cKO::LGI1WT and cKO::LGI1W183R mice (P17). (B) Cumulative plots of mEPSC amplitude. (C) Mean values of mEPSC frequency: 0.23 ± 0.02 Hz (cKO::LGI1WT; n = 7) and 0.22 ± 0.03 Hz (cKO::LGI1W183R ; n = 9), P = 0.88. [file 13578_2023_983_MOESM2_ESM.jpg]

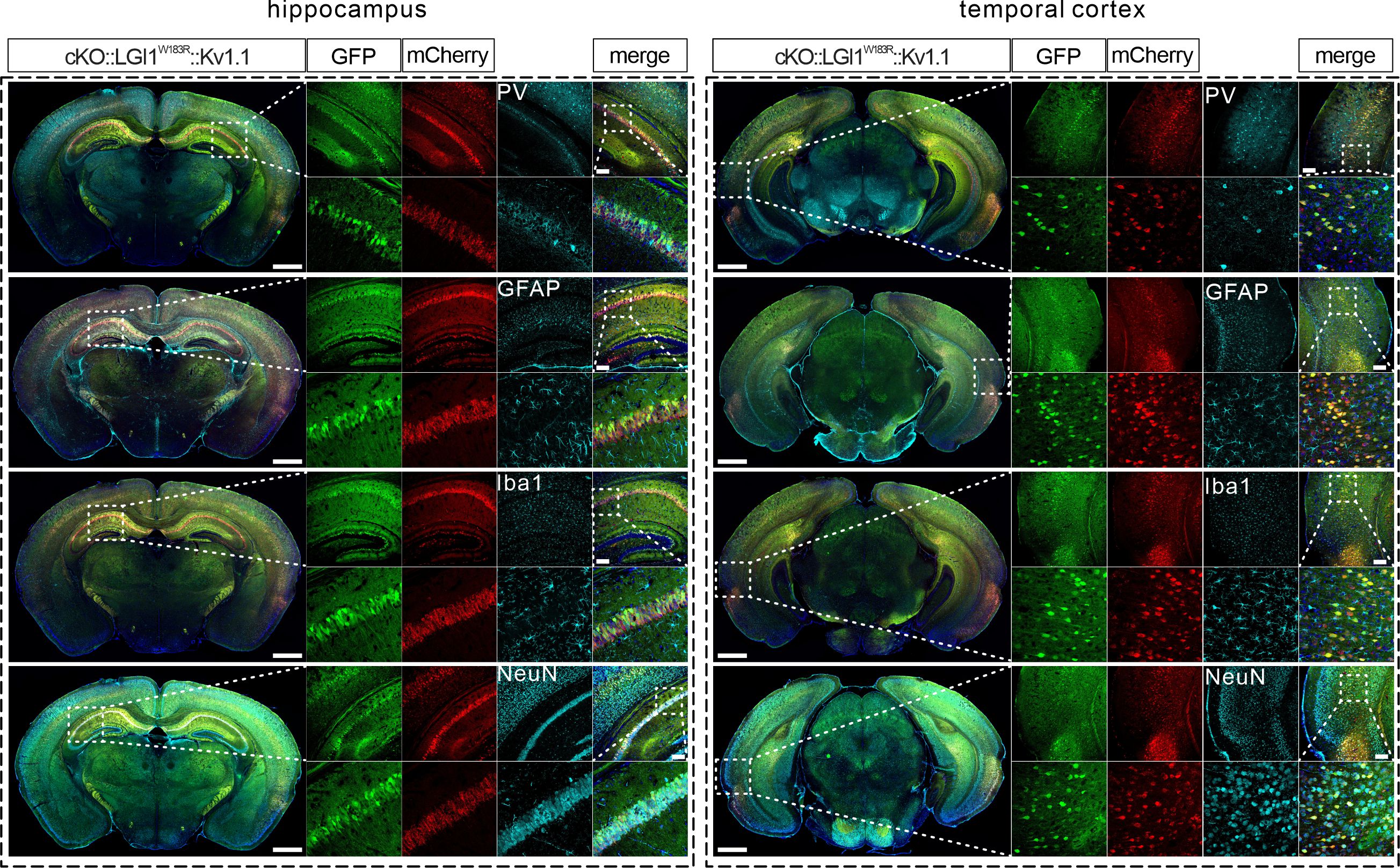

Supplement: Supplementary file 3 — Additional file 3. Figure S3. Restoring Kv1.1 in excitatory neurons does not affect other types of nerve cells. AAV9-DIO-LGI1W183R-GFP and AAV9-DIO-Kv1.1-mCherry were bilaterally injected into the ventricles of cKO mice (P0). Representative images for quadruple fluorescence of GFP, mCherry, individual marker proteins (PV, GFAP, Iba1, and NeuN), and DAPI, show that LGI1W183R is not expressed in PV-positive interneurons, astroglia (GFAP) and microglia (Iba1) in the hippocampus and temporal cortex of cKO mice (P17). Scale bars: 1 mm (whole brain) and 50 μm (magnified). [file 13578_2023_983_MOESM3_ESM.jpg]
