# Supplementary material for: A patient-derived mutation of epilepsy-linked LGI1 increases seizure susceptibility through regulating Kv1.1
Source: Cell Biosci. 2023 Feb 20;13:34. doi: 10.1186/s13578-023-00983-y (PMC9940402; doi:10.1186/s13578-023-00983-y)
Supplement: Supplementary file 4 — Additional file 4. Table S1. The statistics for Fig. 1G, 1H, 1J, and 1K. [file 13578_2023_983_MOESM4_ESM.docx]

**Table S1**

**Statistics for Fig. 1G**

|  | **WT** | **Culture#** | **W183R** | **Culture#** | ***P*** |
| --- | --- | --- | --- | --- | --- |
| **LGI1 mRNA level** | 100 ± 10 | 3 | 98 ± 9 | 3 | 0.40 |

Unpaired *t* test with Welch's correction

**Statistics for Fig. 1H**

|  | **WT** | **Culture#** | **W183R** | **Culture#** | ***P*** |
| --- | --- | --- | --- | --- | --- |
| **LGI1 total protein** | 100 ± 13 | 3 | 98 ± 12 | 3 | 0.43 |

Unpaired *t* test with Welch's correction

**Statistics for Fig. 1J**

|  | **0** | **4** | **8** | **16** | **24** | **Culture#** |
| --- | --- | --- | --- | --- | --- | --- |
| **WT** | 100 | 76 ± 7 | 70 ± 6 | 44 ± 8 | 32 ± 7 | 4 |
| **W183R** | 100 | 92 ± 6 | 88 ± 3 | 75 ± 9 | 66 ± 8 | 4 |
| ***P*** | N/A | 0.2481 | 0.0905 | 0.0019 | 0.0006 |  |

2-way ANOVA followed by Bonferroni's post hoc test

**Statistics for Fig. 1K**

|  | **WT** | **Culture#** | **W183R** | **Culture#** | ***P*** |
| --- | --- | --- | --- | --- | --- |
| **% of WT ub level** | 100 ± 7 | 4 | 101 ± 7 | 4 | 0.39 |

Unpaired *t* test with Welch's correction
