# Supplementary material for: A patient-derived mutation of epilepsy-linked LGI1 increases seizure susceptibility through regulating Kv1.1
Source: Cell Biosci. 2023 Feb 20;13:34. doi: 10.1186/s13578-023-00983-y (PMC9940402; doi:10.1186/s13578-023-00983-y)
Supplement: Supplementary file 5 — Additional file 5. Table S2. The statistics of spontaneous seizures. [file 13578_2023_983_MOESM5_ESM.docx]

**Table S4**

**Statistics for Fig. 4C**

|  | **-70** | **-60** | **-50** | **-40** | **-30** | **-20** | **-10** | **0** | **10** | **20** | **30** | **40** | **Cell# (mouse#)** |
| --- | --- | --- | --- | --- | --- | --- | --- | --- | --- | --- | --- | --- | --- |
| cKO::  LGI1^WT^ | 0.1 ±  0.0 | 0.2 ± 0.04 | 0.4 ± 0.04 | 0.7 ±  0.1 | 1.0 ±  0.1 | 1.5 ±  0.2 | 2.1 ±  0.2 | 3.0 ±  0.4 | 4.1 ±  0.5 | 4.8 ±  0.7 | 5.3 ±  0.7 | 5.9 ±  0.8 | 7 (3) |
| cKO::  LGI1^W183R^ | 0.01 ± 0.0 | 0.2 ±  0.0 | 0.3 ±  0.0 | 0.4 ±  0.0 | 0.6 ±  0.0 | 0.8±  0.1 | 1.2 ±  0.1 | 1.8 ±  0.2 | 2.4 ±  0.2 | 2.9 ±  0.3 | 3.4±  0.3 | 3.7±  0.4 | 6 (3) |
| ***P*** | 0.99 | 0.99 | 0.99 | 0.99 | 0.99 | 0.99 | 0.87 | 0.17 | 0.0061 | 0.0024 | 0.0020 | 0.0003 |  |

2-way ANOVA followed by Bonferroni's post hoc test.

**Statistics for Fig. 4D**

|  | **Mouse type** | **Mean ± SEM** | **Cell# (Mouse#)** | ***P*** |
| --- | --- | --- | --- | --- |
| Activation V_half_ (mV) | cKO::LGI1^WT^ | -17.96 ± 2.17 | 7 (3) | 0.9103 |
|  | cKO::LGI1^W183R^ | -18.51 ± 4.12 | 6 (3) |  |
| Activation slope | cKO::LGI1^WT^ | 19.26 ± 2.21 | 7 (3) | 0.6108 |
|  | cKO::LGI1^W183R^ | 21.19 ± 2.93 | 6 (3) |  |
| Inactivation V_half_ (mV) | cKO::LGI1^WT^ | -49.86 ± 1.51 | 7 (3) | 0.0498 |
|  | cKO::LGI1^W183R^ | -45.39 ± 1.36 | 6 (3) |  |
| Inactivation slope | cKO::LGI1^WT^ | 23.28 ± 2.06 | 7 (3) | 0.8425 |
|  | cKO::LGI1^W183R^ | 22.64 ± 2.36 | 6 (3) |  |

Unpaired *t* test with Welch's correction

**Statistics for Fig. 4H**

|  | **Mouse type** | **Mean ± SEM** | **Cell# (mouse#)** | ***P*** |
| --- | --- | --- | --- | --- |
| Cm (pF) | cKO::LGI1^WT^+DTx-K | 105.6 ± 5.9 | 13 (4) | 0.88 |
|  | cKO::LGI1^W183R^+DTx-K | 103.3 ± 13.8 | 9 (4) |  |
| Rheobase (pA) | cKO::LGI1^WT^+DTx-K | 83.5 ± 7.1 | 13 (4) | 0.90 |
|  | cKO::LGI1^W183R^+DTx-K | 85.0 ± 9.8 | 9 (4) |  |
| RMP (mV) | cKO::LGI1^WT^+DTx-K | -68.8 ± 1.1 | 13 (4) | 0.26 |
|  | cKO::LGI1^W183R^+DTx-K | -67.0 ± 1.0 | 9 (4) |  |
| Threshold (mV) | cKO::LGI1^WT^+DTx-K | -51.1 ± 0.9 | 13 (4) | 0.22 |
|  | cKO::LGI1^W183R^+DTx-K | -52.8 ± 1.0 | 9 (4) |  |
| Half-width (ms) | cKO::LGI1^WT^+DTx-K | 1.4 ± 0.03 | 13 (4) | 0.12 |
|  | cKO::LGI1^W183R^+DTx-K | 1.5 ± 0.04 | 9 (4) |  |
| Amplitude (mV) | cKO::LGI1^WT^+DTx-K | 112.7 ± 2.0 | 13 (4) | 0.53 |
|  | cKO::LGI1^W183R^+DTx-K | 111.1 ± 1.4 | 9 (4) |  |

Unpaired *t* test with Welch's correction

**Statistics for Fig. 4I**

|  | **20** | **40** | **60** | **80** | **100** | **120** | **140** | **160** | **180** | **200** | **Cell# (mouse#)** |
| --- | --- | --- | --- | --- | --- | --- | --- | --- | --- | --- | --- |
| cKO::LGI1^WT^+DTx-K | 0 | 0.92 ± 0.54 | 2.92  ± 1.45 | 6.85  ± 1.41 | 10.15  ± 1.50 | 12.92  ± 1.35 | 14.62  ± 1.23 | 16.08  ± 1.16 | 16.92  ± 1.22 | 18.08  ± 1.32 | 13 (4) |
| cKO::LGI1^W183R^+DTx-K | 0 | 0 | 2.44  ± 1.37 | 5.11  ± 1.70 | 9.56 ± 1.52 | 12.89 ± 1.34 | 14.67 ± 1.24 | 16.11 ± 1.14 | 17.00 ± 1.08 | 17.78  ± 1.14 | 9 (4) |
| ***P*** | 0.99 | 0.99 | 0.99 | 0.98 | 0.99 | 0.99 | 0.99 | 0.99 | 0.99 | 0.99 |  |

2-way ANOVA followed by Bonferroni's post hoc test.

**Statistics for Fig. 4J**

| **1^st^ half-width** | **80** | **100** | **120** | **140** | **160** | **180** | **200** | **Cell# (mouse#)** |
| --- | --- | --- | --- | --- | --- | --- | --- | --- |
| cKO::LGI1^WT^  +DTx-K | 1.45 ± 0.04 | 1.41 ± 0.04 | 1.45 ± 0.04 | 1.48 ± 0.04 | 1.44 ± 0.04 | 1.47 ± 0.04 | 1.46 ± 0.05 | 13 (4) |
| cKO::LGI1^W183R^  +DTx-K | 1.53 ± 0.13 | 1.47 ± 0.10 | 1.48 ± 0.10 | 1.47 ± 0.10 | 1.46 ± 0.10 | 1.46 ± 0.10 | 1.49 ± 0.10 | 9 (4) |
| ***P*** | 0.99 | 0.99 | 0.99 | 0.99 | 0.99 | 0.99 | 0.99 |  |
| **last/1^st^**  **half-width** | **80** | **100** | **120** | **140** | **160** | **180** | **200** | **Cell# (mouse#)** |
| cKO::LGI1^WT^  +DTx-K | 1.05 ± 0.03 | 1.13 ± 0.04 | 1.23 ± 0.06 | 1.42 ± 0.08 | 1.56 ± 0.12 | 1.72 ± 0.15 | 1.89 ± 0.20 | 13 (4) |
| cKO::LGI1^W183R^  +DTx-K | 1.03 ± 0.03 | 1.15 ± 0.03 | 1.31 ± 0.05 | 1.48 ± 0.06 | 1.61 ± 0.08 | 1.79 ± 0.11 | 2.00 ± 0.11 | 9 (4) |
| ***P*** | 0.99 | 0.99 | 0.99 | 0.99 | 0.99 | 0.99 | 0.99 |  |

2-way ANOVA followed by Bonferroni's post hoc test.

**Statistics for Fig. 4L**

|  | **Mouse type** | **Mean ± SEM** | **Cell# (mouse#)** | ***P*** |
| --- | --- | --- | --- | --- |
| First ISI (ms) | cKO::LGI1^WT^+DTx-K | 65.02 ± 7.2 | 9 (5) | 0.99 |
|  | cKO::LGI1^W183R^+DTx-K | 65.2 ± 5.7 | 7 (3) |  |
| CV | cKO::LGI1^WT^+DTx-K | 0.71 ± 0.07 | 9 (5) | 0.42 |
|  | cKO::LGI1^W183R^+DTx-K | 0.78 ± 0.03 | 7 (3) |  |
| CV_2_ | cKO::LGI1^WT^+DTx-K | 0.56 ± 0.09 | 9 (5) | 0.95 |
|  | cKO::LGI1^W183R^+DTx-K | 0.56 ± 0.05 | 7 (3) |  |

Unpaired *t* test with Welch's correction
