# Supplementary material for: A patient-derived mutation of epilepsy-linked LGI1 increases seizure susceptibility through regulating Kv1.1
Source: Cell Biosci. 2023 Feb 20;13:34. doi: 10.1186/s13578-023-00983-y (PMC9940402; doi:10.1186/s13578-023-00983-y)
Supplement: Supplementary file 6 — Additional file 6. Table S3. The statistics for Fig. 3C, 3G, 3H, 3J and 3K. [file 13578_2023_983_MOESM6_ESM.docx]

**Table S2**

**Spontaneous seizures**

| **Mouse type** | **No. mice with seizures (total)** | **No. seizures** | **Age**  **(seizure onset)** | **Seizure frequency per hour** | **Seizure duration (s)** |
| --- | --- | --- | --- | --- | --- |
| cKO::LGI1^WT^ | 0 (5) | 0 | N/A | N/A | N/A |
| cKO::LGI1^W183R^ | 5 (5) | 38 | P16 - P18 | 0.25 - 1 | 45.5 ± 18.7 |
| cKO::LGI1^W183R^::mCherry | 5 (5) | 36 | P16 - P18 | 0.25 - 1 | 47. 9 ± 14.2 |
| cKO::LGI1^W183R^::Kv1.1 | 4 (5) | 25 | P19 - P20 | 0.25 - 0.5 | 35.3 ± 11.2 |
