# Supplementary material for: A patient-derived mutation of epilepsy-linked LGI1 increases seizure susceptibility through regulating Kv1.1
Source: Cell Biosci. 2023 Feb 20;13:34. doi: 10.1186/s13578-023-00983-y (PMC9940402; doi:10.1186/s13578-023-00983-y)
Supplement: Supplementary file 7 — Additional file 7. Table S4. The statistics for Fig. 4C, 4D, 4H, 4I, 4J and 4L. [file 13578_2023_983_MOESM7_ESM.docx]

**Table S5**

**Statistics for Fig. 7A**

|  | **-70** | **-60** | **-50** | **-40** | **-30** | **-20** | **-10** | **0** | **10** | **20** | **30** | **40** | **Cell# mouse#** |
| --- | --- | --- | --- | --- | --- | --- | --- | --- | --- | --- | --- | --- | --- |
| cKO::LGI1^W183R^::  mCherry | 0.0 ± 0.0 | 0.2 ± 0.0 | 0.3 ± 0.0 | 0.4 ± 0.1 | 0.7 ± 0.1 | 0.9 ± 0.1 | 1.3 ± 0.1 | 1.9 ± 0.2 | 2.4 ± 0.3 | 2.8 ± 0.2 | 3.1 ± 0.3 | 3.4 ± 0.3 | 6 (3) |
| cKO::LGI1^W183R^::  Kv1.1 | 0.1 ± 0.0 | 0.2 ± 0.0 | 0.4 ± 0.0 | 0.7 ± 0.1 | 1.1 ± 0.2 | 1.6 ± 0.2 | 2.4 ± 0.2 | 3.0 ± 0.2 | 3.8 ± 0.3 | 4.6 ± 0.4 | 5.2 ± 0.4 | 5.8 ± 0.5 | 6 (3) |
| ***P*** | 0.99 | 0.99 | 0.99 | 0.99 | 0.99 | 0.36 | 0.0090 | 0.0062 | 0.0003 | < 0.0001 | < 0.0001 | < 0.0001 |  |

2-way ANOVA followed by Bonferroni's post hoc test.

**Statistics for Fig. 7B**

|  | **Mouse type** | **Mean ± SEM** | **Cell# (Mouse#)** | ***P*** |
| --- | --- | --- | --- | --- |
| Activation V_half_ (mV) | cKO::LGI1^W183R^::mCherry | -19.9 ± 2.0 | 6 (3) | 0.905 |
|  | cKO::LGI1^W183R^::Kv1.1 | -19.5 ± 1.9 | 6 (3) |  |
| Activation slope | cKO::LGI1^W183R^::mCherry | 20.3 ± 1.9 | 6 (3) | 0.9136 |
|  | cKO::LGI1^W183R^::Kv1.1 | 20.0 ± 2.3 | 6 (3) |  |
| Inactivation V_half_ (mV) | cKO::LGI1^W183R^::mCherry | -44.8 ± 1.3 | 6 (3) | 0.0344 |
|  | cKO::LGI1^W183R^::Kv1.1 | -48.2 ± 0.4 | 6 (3) |  |
| Inactivation slope | cKO::LGI1^W183R^::mCherry | 26.7 ± 2.1 | 6 (3) | 0.7850 |
|  | cKO::LGI1^W183R^::Kv1.1 | 26.1 ± 0.9 | 6 (3) |  |

Unpaired *t* test with Welch's correction.

**Statistics for Fig. 7C**

|  | **Mouse type** | **Mean ± SEM** | **Cell# (Mouse#)** | ***P*** |
| --- | --- | --- | --- | --- |
| Threshold (mV) | cKO::LGI1^W183R^::mCherry | -50.9 ± 1.0 | 12 (3) | 0.0001 |
|  | cKO::LGI1^W183R^::Kv1.1 | -45.2 ± 0.6 | 9 (3) |  |
| Rheobase (pA) | cKO::LGI1^W183R^::mCherry | 110.0 ± 7.6 | 12 (3) | 0.0269 |
|  | cKO::LGI1^W183R^::Kv1.1 | 140.0 ± 9.2 | 9 (3) |  |
| Half-width (ms) | cKO::LGI1^W183R^::mCherry | 1.3 ± 0.0 | 12 (3) | 0.0120 |
|  | cKO::LGI1^W183R^::Kv1.1 | 1.0 ± 0.1 | 9 (3) |  |

Unpaired *t* test with Welch's correction.

**Statistics for other parameters of AP**

|  | **Mouse type** | **Mean ± SEM** | **Cell# (Mouse#)** | ***P*** |
| --- | --- | --- | --- | --- |
| RMP (mV) | cKO::LGI1^W183R^::mCherry | -69.9 ± 0.6 | 12 (3) | 0.23 |
|  | cKO::LGI1^W183R^::Kv1.1 | -71.1 ± 0.8 | 9 (3) |  |
| Cm (pF) | cKO::LGI1^W183R^::mCherry | 118.7 ± 4.3 | 12 (3) | 0.96 |
|  | cKO::LGI1^W183R^::Kv1.1 | 118.0 ± 10.6 | 9 (3) |  |
| Amplitude (mV) | cKO::LGI1^W183R^::mCherry | 112.8 ± 1.4 | 12 (3) | 0.15 |
|  | cKO::LGI1^W183R^::Kv1.1 | 107.9 ± 2.6 | 9 (3) |  |
| dV/dt at 0 mV | cKO::LGI1^W183R^::mCherry | 177.3 ± 1.6 | 12 (3) | 0.72 |
|  | cKO::LGI1^W183R^::Kv1.1 | 182.1 ± 11.3 | 9 (3) |  |
| dV/dt at +20 mV | cKO::LGI1^W183R^::mCherry | -32.2 ± 1.9 | 12 (3) | 0.0022 |
|  | cKO::LGI1^W183R^::Kv1.1 | -40.6 ± 1.5 | 9 (3) |  |
| dV/dt at -40 mV | cKO::LGI1^W183R^::mCherry | -12.0 ± 1.5 | 12 (3) | 0.048 |
|  | cKO::LGI1^W183R^::Kv1.1 | -16.8 ± 1.8 | 9 (3) |  |
| Input resistance  (mV/pA) | cKO::LGI1^W183R^::mCherry | 123.1 ± 4.5 | 12 (3) | 0.86 |
|  | cKO::LGI1^W183R^::Kv1.1 | 121.3 ± 9.1 | 9 (3) |  |

Unpaired *t* test with Welch's correction.

**Statistics for Fig. 7D**

|  | 20 | 40 | 60 | 80 | 100 | 120 | 140 | 160 | 180 | 200 | Cell# mouse# |
| --- | --- | --- | --- | --- | --- | --- | --- | --- | --- | --- | --- |
| cKO::LGI1^W183R^::mCherry | 0 | 0.3 ± 0.3 | 1.4 ± 0.9 | 7.3 ± 0.9 | 12.3 ± 0.9 | 15.2 ± 0.7 | 17.3 ± 0.8 | 18.1 ± 1.0 | 18.3 ± 1.4 | 18.4 ± 1.4 | 12 (3) |
| cKO::LGI1^W183R^::Kv1.1 | 0 | 0 | 0.7 ± 0.4 | 3.4 ± 0.9 | 7.9 ± 1.4 | 12.4 ± 1.1 | 15.7 ± 0.9 | 18.1 ± 0.8 | 20.7 ± 0.9 | 22.4 ± 0.8 | 9 (3) |
| ***P*** | 0.99 | 0.99 | 0.99 | 0.034 | 0.0066 | 0.30 | 0.92 | 0.99 | 0.47 | 0.019 |  |

2-way ANOVA followed by Bonferroni`s post hoc test.

**Statistics for Fig. 7E**

| 1^st^ half-width | **80** | **100** | **120** | **140** | **160** | **180** | **200** | Cell# mouse# |
| --- | --- | --- | --- | --- | --- | --- | --- | --- |
| cKO::LGI1^W183R^::mCherry | 1.4 ± 0.1 | 1.4 ± 0.1 | 1.3 ± 0.1 | 1.3 ± 0.1 | 1.3 ± 0.1 | 1.3 ± 0.1 | 1.3 ± 0.1 | 12 (3) |
| cKO::LGI1^W183R^::Kv1.1 | 1.1 ± 0.1 | 1.0 ± 0.1 | 1.0 ± 0.1 | 1.0 ± 0.1 | 1.0 ± 0.1 | 1.0 ± 0.1 | 1.0 ± 0.1 | 9 (3) |
| ***P*** | 0.0007 | 0.0021 | 0.0057 | 0.0055 | 0.012 | 0.016 | 0.010 |  |
| last/1^st^  half-width | **80** | **100** | **120** | **140** | **160** | **180** | **200** | Cell# mouse# |
| cKO::LGI1^W183R^::mCherry | 1.2 ± 0.0 | 1.3 ± 0.1 | 1.6 ± 0.1 | 1.7 ± 0.2 | 1.9 ± 0.2 | 2.2 ± 0.2 | 2.3 ± 0.2 | 12 (3) |
| cKO::LGI1^W183R^::Kv1.1 | 1.1 ± 0.0 | 1.2 ± 0.1 | 1.1 ± 0.0 | 1.1 ± 0.0 | 1.3 ± 0.1 | 1.4 ± 0.1 | 1.5 ± 0.1 | 9 (3) |
| ***P*** | 0.99 | 0.99 | 0.30 | 0.038 | 0.013 | 0.0015 | 0.0022 |  |

2-way ANOVA followed by Bonferroni's post hoc test.

**Statistics for Fig. 7G**

|  | **Mouse type** | **Mean ± SEM** | **Cell# (mouse#)** | ***P*** |
| --- | --- | --- | --- | --- |
| First ISI (ms) | cKO::LGI1^W183R^::mCherry | 56.4 ± 8.0 | 7 (3) | 0.0049 |
|  | cKO::LGI1^W183R^::Kv1.1 | 22.9 ± 2.5 | 9 (4) |  |
| CV | cKO::LGI1^W183R^::mCherry | 0.5 ± 0.1 | 7 (3) | 0.0262 |
|  | cKO::LGI1^W183R^::Kv1.1 | 0.4 ± 0.0 | 9 (4) |  |
| CV_2_ | cKO::LGI1^W183R^::mCherry | 0.6 ± 0.1 | 7 (3) | 0.0192 |
|  | cKO::LGI1^W183R^::Kv1.1 | 0.3 ± 0.0 | 9 (4) |  |

Unpaired *t* test with Welch’s correction.

**Statistics for Fig. 7H**

| **Spike number** | **1** | **2** | **3** | **4** | **5** | **6** | **7** | Cell# mouse# |
| --- | --- | --- | --- | --- | --- | --- | --- | --- |
| cKO::LGI1^W183R^::mCherry | -1.4 ± 0.2 | -4.9 ± 0.8 | -6.1 ± 1.3 | -2.6 ± 0.2 | -2.6 ± 0.3 | -2.6 ± 0.4 | -3.0 ± 0.4 | 7 (3) |
| cKO::LGI1^W183R^::Kv1.1 | -0.6 ± 0.2 | -1.5 ± 0.2 | -1.9 ± 0.3 | -1.9 ± 0.4 | -1.9 ± 0.4 | -1.9 ± 0.3 | -1.9 ± 0.5 | 9 (4) |
| ***P*** | 0.82 | 0.0033 | < 0.0001 | 0.94 | 0.99 | 0.96 | 0.83 |  |

2-way ANOVA followed by Bonferroni's post hoc test.
