# Supplementary material for: A patient-derived mutation of epilepsy-linked LGI1 increases seizure susceptibility through regulating Kv1.1
Source: Cell Biosci. 2023 Feb 20;13:34. doi: 10.1186/s13578-023-00983-y (PMC9940402; doi:10.1186/s13578-023-00983-y)
Supplement: Supplementary file 8 — Additional file 8. Table S5. The statistics for Fig. 7A, 7B, 7C, 7D, 7E, 7G and 7H. [file 13578_2023_983_MOESM8_ESM.docx]

**Table S3**

**Statistics for Fig. 3C**

|  | **Mouse type** | **Mean ± SEM** | **Cell# (mouse#)** | ***P*** |
| --- | --- | --- | --- | --- |
| **Cm (pF)** | cKO::LGI1^WT^ | 111.1 ± 8.1 | 17 (5) | 0.87 |
|  | cKO::LGI1^W183R^ | 108.6 ± 12.0 | 9 (3) |  |
| **Rheobase (pA)** | cKO::LGI1^WT^ | 124.4 ± 6.1 | 17 (5) | 0.0003 |
|  | cKO::LGI1^W183R^ | 88.9 ± 5.7 | 9 (3) |  |

Unpaired *t* test with Welch's correction

**Statistics for Fig. 3E**

|  | **Mouse type** | **Mean ± SEM** | **Cell# (mouse#)** | ***P*** |
| --- | --- | --- | --- | --- |
| **RMP (mV)** | cKO::LGI1^WT^ | -72.0 ± 0.7 | 17 (5) | 0.077 |
|  | cKO::LGI1^W183R^ | -68.7 ± 0.9 | 9 (3) |  |
| **Threshold (mV)** | cKO::LGI1^WT^ | -43.5 ± 1.1 | 17 (5) | < 0.0001 |
|  | cKO::LGI1^W183R^ | -51.1 ± 0.6 | 9 (3) |  |
| **Half-width (ms)** | cKO::LGI1^WT^ | 1.1 ± 0.0 | 17 (5) | 0.0002 |
|  | cKO::LGI1^W183R^ | 1.4 ± 0.1 | 9 (3) |  |
| **Amplitude (mV)** | cKO::LGI1^WT^ | 115.1 ± 1.1 | 17 (5) | 0.17 |
|  | cKO::LGI1^W183R^ | 110.8 ± 2.7 | 9 (3) |  |
| **dV/dt at 0 mV** | cKO::LGI1^WT^ | 177.8 ± 6.8 | 17 (5) | 0.75 |
|  | cKO::LGI1^W183R^ | 181.7 ± 9.8 | 9 (3) |  |
| **dV/dt at +20 mV** | cKO::LGI1^WT^ | -39.7 ± 1.1 | 17 (5) | < 0.0001 |
|  | cKO::LGI1^W183R^ | -30.3 ± 1.0 | 9 (3) |  |
| **dV/dt at -40 mV** | cKO::LGI1^WT^ | -16.8 ± 1.2 | 17 (5) | 0.038 |
|  | cKO::LGI1^W183R^ | -11.9 ± 1.7 | 9 (3) |  |

Unpaired *t* test with Welch's correction

**Statistics for Fig. 3G**

|  | **20** | **40** | **60** | **80** | **100** | **120** | **140** | **160** | **180** | **200** | **Cell# (mouse#)** |
| --- | --- | --- | --- | --- | --- | --- | --- | --- | --- | --- | --- |
| cKO::LGI1^WT^ | 0 | 0 | 0.06  ± 0.06 | 3.7  ± 0.5 | 10.2  ± 0.8 | 14.8  ± 0.8 | 17.6  ± 0.7 | 20.0  ± 0.7 | 22.0  ± 0.7 | 23.8  ± 0.7 | 17 (5) |
| cKO::LGI1^W183R^ | 0 | 0.22 ± 0.22 | 2.89  ± 1.15 | 6.78  ± 1.32 | 11.33 ± 1.03 | 14.56 ± 0.69 | 16.78 ± 0.60 | 18.22 ± 0.72 | 19.22 ± 0.66 | 20.2  ± 0.8 | 9 (3) |
| ***P*** | 0.99 | 0.99 | 0.043 | 0.019 | 0.99 | 0.99 | 0.99 | 0.81 | 0.05 | 0.003 |  |

2-way ANOVA followed by Bonferroni's post hoc test.

**Statistics for Fig. 3H**

| **1^st^ half-width** | **80** | **100** | **120** | **140** | **160** | **180** | **200** | **Cell# mouse#** |
| --- | --- | --- | --- | --- | --- | --- | --- | --- |
| cKO::LGI1^WT^ | 1.1 ± 0.04 | 1.1 ± 0.03 | 1.0 ± 0.03 | 1.0 ± 0.02 | 1.0 ± 0.03 | 1.0 ± 0.03 | 1.0 ± 0.03 | 17 (5) |
| cKO::LGI1^W183R^ | 1.4 ± 0.04 | 1.4 ± 0.04 | 1.4 ± 0.03 | 1.3 ± 0.05 | 1.3 ± 0.05 | 1.3 ± 0.05 | 1.4 ± 0.05 | 9 (3) |
| ***P*** | < 0.0001 | < 0.0001 | < 0.0001 | < 0.0001 | < 0.0001 | < 0.0001 | < 0.0001 |  |
| **last/1^st^**  **half-width** | **80** | **100** | **120** | **140** | **160** | **180** | **200** | **Cell# mouse#** |
| cKO::LGI1^WT^ | 1.0 ± 0.02 | 1.1 ± 0.03 | 1.1 ± 0.01 | 1.2 ± 0.04 | 1.3 ± 0.05 | 1.4 ± 0.05 | 1.5 ± 0.05 | 17 (5) |
| cKO::LGI1^W183R^ | 1.1 ± 0.02 | 1.2 ± 0.03 | 1.3 ± 0.03 | 1.4 ± 0.04 | 1.5 ± 0.04 | 1.6 ± 0.05 | 1.7 ± 0.04 | 9 (3) |
| ***P*** | 0.70 | 0.35 | 0.0554 | 0.0203 | 0.0079 | 0.0275 | 0.0023 |  |

2-way ANOVA followed by Bonferroni's post hoc test.

**Statistics for Fig. 3J**

|  | **Mouse type** | **Mean ± SEM** | **Cell# (mouse#)** | ***P*** |
| --- | --- | --- | --- | --- |
| First ISI (ms) | cKO::LGI1^WT^ | 16.7 ± 1.2 | 8 (7) | 0.0016 |
|  | cKO::LGI1^W183R^ | 48.2 ± 6.5 | 8 (3) |  |
| CV | cKO::LGI1^WT^ | 0.4 ± 0.1 | 8 (7) | 0.0035 |
|  | cKO::LGI1^W183R^ | 0.6 ± 0.1 | 8 (3) |  |
| CV_2_ | cKO::LGI1^WT^ | 0.2 ± 0.0 | 8 (7) | 0.0001 |
|  | cKO::LGI1^W183R^ | 0.5 ± 0.0 | 8 (3) |  |

Unpaired *t* test with Welch's correction

**Statistics for Fig. 3K**

| **Spike number** | **1** | **2** | **3** | **4** | **5** | **6** | **7** | **Cell# mouse#** |
| --- | --- | --- | --- | --- | --- | --- | --- | --- |
| cKO::LGI1^WT^ | -0.6 ± 0.3 | -1.6 ± 0.7 | -2.1 ± 1.0 | -2.0 ± 1.0 | -2.0 ± 1.0 | -1.9 ± 0.9 | -2.1 ± 1.0 | 8 (7) |
| cKO::LGI1^W183R^ | -1.1 ± 0.4 | -4.6 ± 0.6 | -5.1 ± 0.7 | -2.9 ± 0.4 | -2.9 ± 0.4 | -3.0 ± 0.4 | -2.7 ± 0.4 | 8 (3) |
| ***P*** | 0.99 | 0.011 | 0.011 | 0.92 | 0.91 | 0.85 | 0.99 |  |

2-way ANOVA followed by Bonferroni's post hoc test.
