# Supplementary material for: A patient-derived mutation of epilepsy-linked LGI1 increases seizure susceptibility through regulating Kv1.1
Source: Cell Biosci. 2023 Feb 20;13:34. doi: 10.1186/s13578-023-00983-y (PMC9940402; doi:10.1186/s13578-023-00983-y)
Supplement: Supplementary file 11 — Additional file 11. Proband clinical information. [file 13578_2023_983_MOESM11_ESM.pdf]

Name:	*Name hided*	SampleID:	R22010414	Sample Type:	Blood
Birthday:	1999-12	FamilyID:		Receive date:	2017-05-14
Gender:	Male	Member:	R22010655(sibling)	Report date:	2017-07-14
Hospital:	The First Affiliated Hospital, Zhejiang University School of Medicine		R22010415(niece)	Version:	v2.0
Physician:	Wang,Kang	Age:	17	Ethinic:	NA

Test Project and result

Symptoms and clinical diagnosis:

Medical history: The patient suffered from recurrent unconsciousness, convulsions (nearly 2 years) and aura of tinnitus since 16-year-old. The EEG showed a spike in the right temporofrontal region, and the MRI scan of the head was negative. Family History: Father had similar symptoms, which onset at 15 year old.
Clinical diagnosis: focal epilepsy.

Test type:

Trio Whole Exome Testing: Whole exome capture and sequencing of patient and parental genomic DNA.

Conclusion: Inconclusive

Result:

Relative Finding: Variants of uncertain significance(VUS) have been identified to be completely/strongly associated with the phenotype

Gene Symbol	Position	HGVScp	Zygosity	Disease	Inheritance	Origin	Classification
LGI1	chr10:95552543	NM_005097.2:c.547T>C (p.Trp183Arg)	heterozygosity	Epilepsy, familial temporal lobe, 1 [MIM600512]	AD	Paternal	VUS

Description:

Disease-causing variants in the *LGI1* gene likely leads to patients suffering from familial temporal lobe epilepsy type 1 (Epilepsy, familial temporal lobe, 1, ETL1) [MIM 600512]. It has been known as autosomal dominant partial epilepsy with auditory features (ADPEAF). The inheritance mode of the disease is autosomal dominant inheritance, and the carriers having the *LGI1* mutation might not suffer from the symptom of the disease[incomplete penetrance] (PMID: 18711109, etc.). The onset age range of the disease is wide (4 to 50 years), usually present during adolescence or early adulthood. The major medical symptom of ETL1 is focal seizures with auditory aura symptoms (such as tinnitus, etc.), accompanied by aphasia. Most of the patients have mild disease progression without involving other systems, and antiepileptic treatment is effective for the patients.

The *LGI1*:c.547T>C (p.Trp183Arg) variant identified in this patient has not been reported in literature and population databases. Both the patient and the affected father carry this variant. Based on abovementioned evidence, this variant is defined as a variant of unknown clinical significance.

It is recommended that clinicians conduct clinical examinations of the patient and father in combination with the characteristics of ETL1. It is recommended to further comfirm the sequencing of the gene locus in paternal family members and review the clinical examination or medical history. By combining the information of the mutation carried in the family and clinical manifestations of family members, clinician could find more disease-causing evidence to explain the test results. Genetic counseling is recommended for the paternal family members of the patient.

Name:	*Name hided*	SampleID:	R22010414	Sample Type:	Blood
Birthday:	1999-12	FamilyID:		Receive date:	2017-05-14
Gender:	Male	Member:	R22010655(sibling)	Report date:	2017-07-14
Hospital:	The First Affiliated Hospital, Zhejiang University School of Medicine		R22010415(niece)	Version:	v2.0
Physician:	Wang,Kang	Age:	17	Ethinic:	NA

Other Finding: P/LP variants or variants of uncertain significance(VUS) have been identified to be partially associated with the phenotype or P/LP variants have been identified to be completely/strong associated with the phenotype but not supporting the inheriationce of the pheynotype:

Gene Symbol	Position	HGVScp	Zygosity	Disease	Inheritance	Origin	Classification
SZT2	chr1:43885465-43885469	NM_015284.3:c.940_944delinsGCAA G>A (p.Lys314Alafs*25)	het	Developmental and epileptic encephalopathy 18 [MIM: 615476]	AR	Paternal	LP
CHRNA4	chr20: 61981213	NM_000744.6:c.1550C>T (p.Ser517Leu)	het	Epilepsy, nocturnal frontal lobe, 1 [MIM:600513]	AD	Paternal	VUS

Description:

Disease-causing variants in the SZT2 gene is likely assocated with Epileptic encephalopathy, early infantile, 18 (EIEE18) [MIM: 615476] in a patient. The disease is considered to be inherited in an autosomal recessive manner. The medical manifestations of EIEE18 are postnatal psychomotor retardation, facial deformity, early-onset dystrophy and so on. The patients with epilepsy whose brain imaging usually shows some features such as thickenness of the corpus callosum and persistent cavum septum pellucidum. This patient's epilepsy occurrence is partially concordant with the partial symptom of EIEE18.

The *SZT2*:c.940_944delinsGCAA (p.Lys314Alafs*25) mutation carried by this patient is a frameshift mutation in the coding region of the SZT2 gene, which theoretically leads to mRNA bearing with Premature termination codons(PTC). There are papers reporting the presence of the multiple null variants downstream this mutation, suggesting that the missing part of the protein still has an important effect on the protein function. This variant has not been reported in the literature or population databases. The variant was also detected in the symptomatic father in this patient's family. Based on the abovementioned evidence, this variant is currently defined as a likely pathogenic variant.

According to the onset age, clinical manifestations, and inheritance patterns of the patient and father both carrying this variant are not highly consistent with the disease, it is recommended to confirm based on other clinical examinations combining with the clinical features of EIEE18. If other clinical test result could not provide evidence to supporting the patient suffering from this dease, the variant may not be the cause of epilepsy in this family. It should be noted that this result does not rule out the co-effect of multiple genes.

Suggestion:

It is recommended that clinicians refer to this test report, combine the clinical manifestations of the patient, improve the corresponding examination, formulate a treatment plan, and conduct corresponding genetic counseling.

Declaration:

The amount of high-throughput sequencing data is huge, and the analysis of the results relies on clinically provided medical history information, existing database information, and published literature. The experimental performance characteristics were confirmed by Clearcode Biotechnology Laboratory and have not been approved by the State Food and Drug Administration. If you have any questions about the test results, please contact the laboratory (Tel: 021-50461580). Since specimens are kept for a certain period of time, please submit an application for re-examination within 20 days from the report date.

In view of the rapid progress of disease-causing gene research, our laboratory will pay attention to the follow-up data analysis and interpretation of the detected cases. If the clinical significance of some specific variants may not be clear when this analysis is performed, it can be requested through the sending doctor 3 months after the issuance of this report, and the exome sequencing data can be re-analyzed and updated regularly.

Restriction:

1. Using the whole exome capture high-throughput sequencing technology, only the coding region of the gene is sequenced, and the average data coverage is 90-110X. This method cannot completely cover repetitive regions, GC-rich regions, pseudogene regions, etc.
2. This method is suitable for point variation and small fragment insertion and deletion variation. It is not suitable for the detection of large fragment copy number variation, dynamic variation and complex recombination and other special types of variation, nor is it suitable for the detection of genomic structural variation and large fragment insertion variation. and variants located in gene regulatory regions and intron regions.
3. For non-clear pathogenic variants, please combine with clinical, and should not be directly used as the basis for clinical decision-making.
4. Not all identified variants will be reported in this test, only variants in known disease-causing genes with evidence of disease-causing variants will be reported. Benign or suspected benign variants will not be reported.
5. The DNA used in this method is derived from the blood or somatic cells of the subject, not from germ cells, so interpretation bias caused by chimerism cannot be ruled out.
6. This test is based on the assumption that both the parents of the child are biological parents, and this report does not involve consanguinity.
7. Only pathogenic variants consistent with clinical symptoms at the time of application are reported in this test result. Gene variants for tumors, non-onset, complex diseases, etc., are not within the scope of this report.
8. In view of the current limitations of human understanding of the disease, the purpose of DNA sequence analysis is to understand the cause of the disease or assess the genetic risk. If no pathogenic variant of a specific gene is detected, it is a negative result, but this does not Excluding the possibility of a certain disease, there are still other unknown genes or gene variants that are difficult to detect or cannot be identified or non-genetic factors involved.
9. Due to the current lack of understanding of certain genes, the detected specific gene variants, in some cases, may not be the pathogenic gene variants that cause the disease, and further verification and research are required.
10. This testing technology and related instruments are not routine clinical testing items, and are currently mainly used for assisting clinical diagnosis or scientific research and other related purposes. In addition, with any genetic testing experiment, there is a very small (<5%) risk of error. The results of this test need to be comprehensively judged by clinicians in combination with all aspects of the situation.

Appendix

Phenotype List(Human Phenotype Ontology):

- HP:0001250: Seizures
- HP:0000364: Hearing abnormality

Methods:

High-throughput sequencing uses the Illumina HiSeq platform to align with the human reference genome sequence, and 95% of the target capture regions have a sequencing depth greater than 20X. The analysis was performed using Clinical Sequence Analyzer (CSA) software, which was used to discover disease-causing genes and clinically significant genetic variants associated with patient phenotypes.

Sequence:

Using Agilent's SureSelect Human All Exon V5 kit, Illumina Cluster, and SBS, the average sequencing depth of the target region was >=90X for exome sequencing, with 95% of the target sequences having a sequencing depth of more than 20X. All sequenced fragments were base-called, and the Burrows-Wheeler alignment algorithm (BWA) was used to align with the UCSC hg19 human reference genome sequence, and GATK (Genomic Analysis Tool Kit) was used to identify variants. This assay was established.

Secondary analysis:

The sequenced fragments were aligned with the UCSC hg19 reference genome by BWA software (version: 0.7.9a) [Li et al]. The alignment results were analyzed by using Picard (version: 1.115) (or Biobambam, v2.0.8) software to remove PCR repeats, and then for downstream data analysis. Variation detection was performed using GATK (Genome Analysis Tool Kit, version: v3.2) software [McKenna A. et al, Genome Res. 2010 Sep; 20(9)], including: Base quality score correction, InDels position and quality Score-corrected, SNVs and InDels variant discovery and typing [DePristo M. et al. Nature Genetics. 2011 Apr; 43(5)].

Annotation and Selection of variants:

Variations were annotated using a process developed in clearcode (WuXi NextCODE). In addition, the sequencing depth of each base was obtained from all genome sequencing data. Variants were annotated using VEP software (Variant Effect Predictor, Ensembl 73), in which functional coding region and splice site variants will be analyzed in the next step, mainly including loss-of-function variants (variants with stop codons, frameshift variants) and critical splice site variants), missense variants, non-frameshift deletions/insertions. These variants were all predicted to have high or high functional impact. Three major databases of known or suspected pathogenic variants, including ClinVar, OMIM and HGMD, will be used to screen for known pathogenic variants, along with tools to predict the function of missense variants and annotation of noncoding regulatory sequences and many more. Large-scale population-based sequencing databases were used to exclude variants with higher frequencies in the normal population. It should be noted that each public or private database used in the variant annotation process is regularly updated, so when analyzing the data in this case, there is a possibility that the latest literature evidence may not be synchronized to the relevant database, so patients with negative test results can apply for re-analysis. Interpretation, please contact customer service for details.

Curation and Classification:

Each variant will be evaluated using developed and validated Clinical Sequence Analyzer (CSA) software, and will be evaluated in accordance with the Standards and Guidelines for Interpretation of Sequence Variants published by the American College of Medical Genetics and Genomics (ACMG). variants are classified. Sequence variants use HGVS nomenclature.
