# Supplementary material for: A patient-derived mutation of epilepsy-linked LGI1 increases seizure susceptibility through regulating Kv1.1
Source: Cell Biosci. 2023 Feb 20;13:34. doi: 10.1186/s13578-023-00983-y (PMC9940402; doi:10.1186/s13578-023-00983-y)
Supplement: Supplementary file 12 — Additional file 12. Proband family sequencing result. [file 13578_2023_983_MOESM12_ESM.pdf]

YD17080973 *(name hided)* Sanger Sequencing results

1. Mutation identification and genetic validation:

Gene	Mutation position	Grandfather	Grandmother	Great Uncle
LGI1	c.547T>C chr10:95552543	heterozygous	No mutation	No mutation

2. Interpretation of results:

The proband's grandfather had a heterozygous LGI1 c.547T＞C mutation, while his grandmather and great uncle had no mutations. LGI1 is inherited in an autosomal dominant pattern; if this mutation is pathogenic, it may be considered the most likely causative variant of this patient. Further analysis in combination with his clinical conditions is required.

3. Results:

The proband's grandfather (SH17089794), had a heterozygous c.547T>C mutation at chr10:95552543.

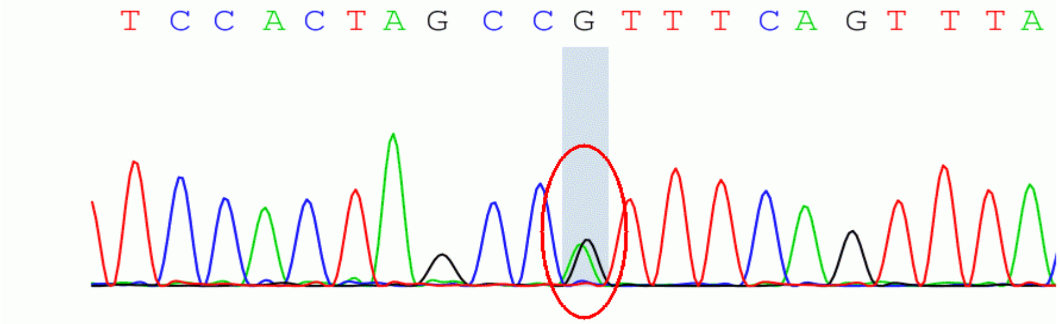

The proband's grandmother (SH17089795), had no mutation at chr10:95552543

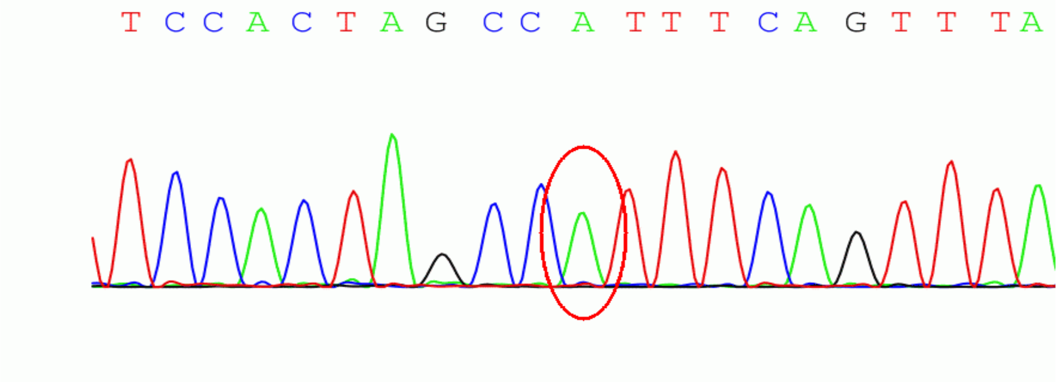

The proband's great uncle (SH17089796), had no mutation at chr10:95552543

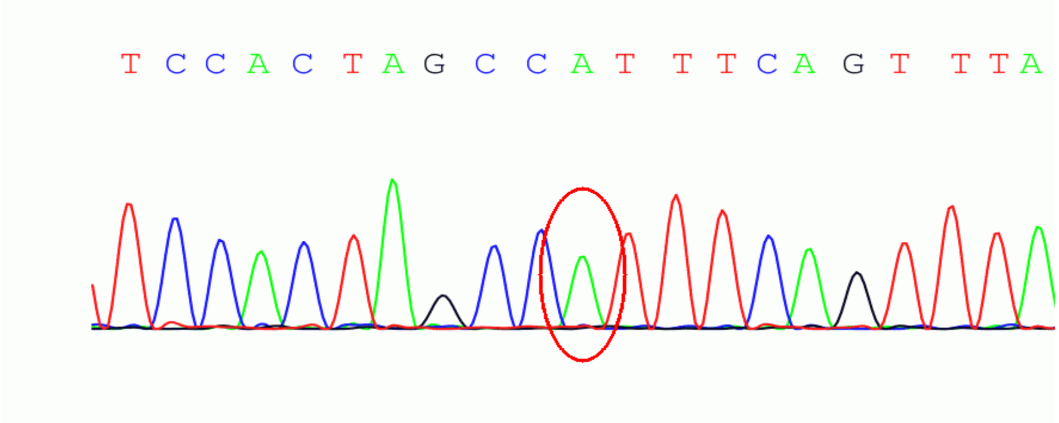

Description of testing techniques and limitations:

1. The molecular genetic testing and analysis of this project is carried out for a specific sample, and we are only responsible for this sample.
2. The above conclusions are laboratory results and the report is only for clinical reference, not as the final diagnosis. Please consult a specialist or professional genetic counselor for the analysis results.
3. The significance of this test is to find potential cause of the disease, so as to assist clinical diagnosis. Due to the complexity and progression of the disease and the limitations of each technique, this test only provides the variation within the detection range.
4. Please contact us within 7 working days of receiving the results if you have any doubts about the results!
